# Targeting Squalene Epoxidase Confers Metabolic Vulnerability and Overcomes Chemoresistance in HNSCC

**DOI:** 10.1002/advs.202206878

**Published:** 2023-07-25

**Authors:** Xinyuan Zhao, Bing Guo, Wenjuan Sun, Jinhua Yu, Li Cui

**Affiliations:** ^1^ Stomatological Hospital School of Stomatology Southern Medical University Guangzhou 510280 China; ^2^ Department of Dentistry the First Affiliated Hospital Sun Yat‐sen University Guangzhou 510080 China; ^3^ Department of Stomatology The Third Affiliated Hospital Sun Yat‐sen University Guangzhou 510630 China; ^4^ Department of Endodontics Jiangsu Key Laboratory of Oral Diseases Affiliated Hospital of Stomatology Nanjing Medical University Nanjing 210029 China; ^5^ Division of Oral Biology and Medicine School of Dentistry University of California Los Angeles Los Angeles CA 90095 USA

**Keywords:** cholesterol, cisplatin resistance, c‐Myc, lipid raft, squalene epoxidase

## Abstract

Cisplatin resistance poses a substantial hurdle in effectively treating head and neck squamous cell carcinoma (HNSCC). Utilizing multiple tumor models and examining an internal HNSCC cohort, squalene epoxidase (SQLE) is pinpointed as a key driver of chemoresistance and tumorigenesis, operating through a cholesterol‐dependent pathway. Comprehensive transcriptomic analysis reveals that SQLE is essential for maintaining c‐Myc transcriptional activity by stabilizing the c‐Myc protein and averting its ubiquitin‐mediated degradation. Mechanistic investigation demonstrates that SQLE inhibition diminishes Akt's binding affinity to lipid rafts via a cholesterol‐dependent process, subsequently deactivating lipid raft‐localized Akt, reducing GSK‐3*β* phosphorylation at S9, and increasing c‐Myc phosphorylation at T58, ultimately leading to c‐Myc destabilization. Importantly, employing an *Sqle* conditional knockout mouse model, SQLE's critical role in HNSCC initiation and progression is established. The preclinical findings demonstrate the potent synergistic effects of combining terbinafine and cisplatin in arresting tumor growth. These discoveries not only provide novel insights into the underlying mechanisms of SQLE‐mediated cisplatin resistance and tumorigenesis in HNSCC but also propose a promising therapeutic avenue for HNSCC patients unresponsive to conventional cisplatin‐based chemotherapy.

## Graphical abstract

This study delineates the crucial role of squalene epoxidase (SQLE) in driving chemoresistance and tumorigenesis in head and neck squamous cell carcinoma. Mechanistically, SQLE inhibition impedes Akt's lipid raft binding by diminishing cholesterol levels within lipid rafts, subsequently modulating GSK‐3*β* and c‐Myc phosphorylation. This culminates in ubiquitin‐mediated c‐Myc degradation, unveiling a promising therapeutic strategy targeting SQLE for overcoming chemoresistance.

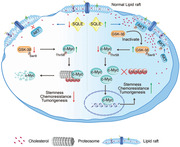

## 1 Introduction

Head and neck malignancies are the sixth most common cancer globally, and among these, head and neck squamous cell carcinoma (HNSCC) represents over 90% of all head and neck cancer cases.^[^ ^1^ ^]^ HNSCC arises from the malignant transformation of mucosal epithelial cells in the oral cavity, oropharynx, hypopharynx, and larynx. Notable risk factors encompass smoking, alcohol abuse, human papillomavirus (HPV) infection, betel nut chewing, and inadequate intake of vitamins and trace elements.^[^ ^2^, ^3^, ^4^ ^]^ Due to the abundant blood supply, dense lymphatic tissue distribution, and frequent local movements in the head and neck region, HNSCC's rapid progression is predisposed to invasion and metastasis. Although there have been significant advances in surgical resection, radiotherapy, chemotherapy, molecular targeted therapy, and immunotherapy, the five‐year survival rate for HNSCC patients remains unchanged at ≈60%.^[^ ^5^, ^6^ ^]^ The primary contributors to the unfavorable clinical outcomes of HNSCC include delayed diagnosis, therapy resistance, and a dearth of specific biomarkers and effective therapeutic targets.

Cisplatin‐based concurrent chemoradiotherapy is the gold standard for treating locally advanced HNSCC.^[^ ^7^, ^8^ ^]^ However, extended cisplatin exposure frequently fosters drug resistance in tumor cells, culminating in treatment failure and diminished prognosis.^[^ ^9^ ^]^ As platinum compounds, exemplified by cisplatin, are the predominant first‐line chemotherapy agents in HNSCC clinical management, surmounting cisplatin resistance is imperative for augmenting therapeutic efficacy. Thus, a comprehensive understanding of the molecular mechanisms driving cisplatin resistance will lay a pivotal groundwork for the development of innovative therapeutic targets in HNSCC.

Cellular metabolism encompasses a complex network of biochemical reactions that convert metabolites to fulfill vital biological functions, thereby maintaining cellular homeostasis. A hallmark of cancer, metabolic reprogramming, allows tumor cells to rewire metabolic processes, fostering tumorigenesis and chemoresistance.^[^ ^10^, ^11^ ^]^ Notably, lipid metabolism dysregulation is among the most significant metabolic alterations in cancer. Cholesterol, an indispensable component of cell membranes and a precursor for steroid hormones, plays a pivotal role in cancer progression.^[^ ^12^ ^]^ Reprogramming cholesterol metabolism not only impacts the malignant behavior of cancer cells but also shapes the antitumor activities of immune cells within the tumor microenvironment.^[^ ^13^, ^14^, ^15^ ^]^ Although altered cholesterol levels have been observed in HNSCC tissues and cholesterol‐lowering drugs appear to enhance treatment efficacy,^[^ ^16^, ^17^, ^18^ ^]^ the potential involvement of cholesterol and cholesterol‐related metabolic enzymes in HNSCC cisplatin resistance remains an unresolved issue.

In this study, the expression of cholesterol‐related metabolic enzymes was initially assessed in chemoresistant xenograft models developed in vivo. A notable increase in squalene epoxidase (SQLE) levels was observed, which were transcriptionally upregulated by the *β*‐catenin/TCF4 complex. We subsequently utilize an internal HNSCC patient cohort, unbiased genome‐wide transcriptomic analysis, patient‐derived xenograft (PDX) models, and conditional knockout models to decipher the molecular mechanisms of SQLE in mediating tumorigenesis and cisplatin resistance in HNSCC. Our investigation reveals that SQLE is indispensable for preserving cholesterol homeostasis in lipid rafts of HNSCC cells, facilitating Akt‐lipid raft interaction by augmenting cholesterol content within lipid rafts, which in turn activates lipid raft‐associated Akt signaling. This activation leads to enhanced phosphorylation of GSK3*β* at Ser9, deactivating GSK3*β*, and diminishing c‐Myc phosphorylation on Thr58, effectively stabilizing c‐Myc by inhibiting the ubiquitin‐proteasome pathway. The accumulation of c‐Myc underscores SQLE's tumor‐promoting role in HNSCC, and targeting c‐Myc circumvents SQLE‐mediated cisplatin resistance. Notably, the combination of cisplatin and SQLE inhibitor terbinafine exhibits a potent synergistic effect in treating HNSCC in both PDX and orthotopic models. Altogether, these findings propose promising treatment strategies for cisplatin‐resistant HNSCC patients by targeting SQLE.

## 2 Results

### 2.1 Upregulated SQLE Associated with Cisplatin Resistance and Unfavorable Clinical Outcomes in HNSCC Patients

**Figure 1:**
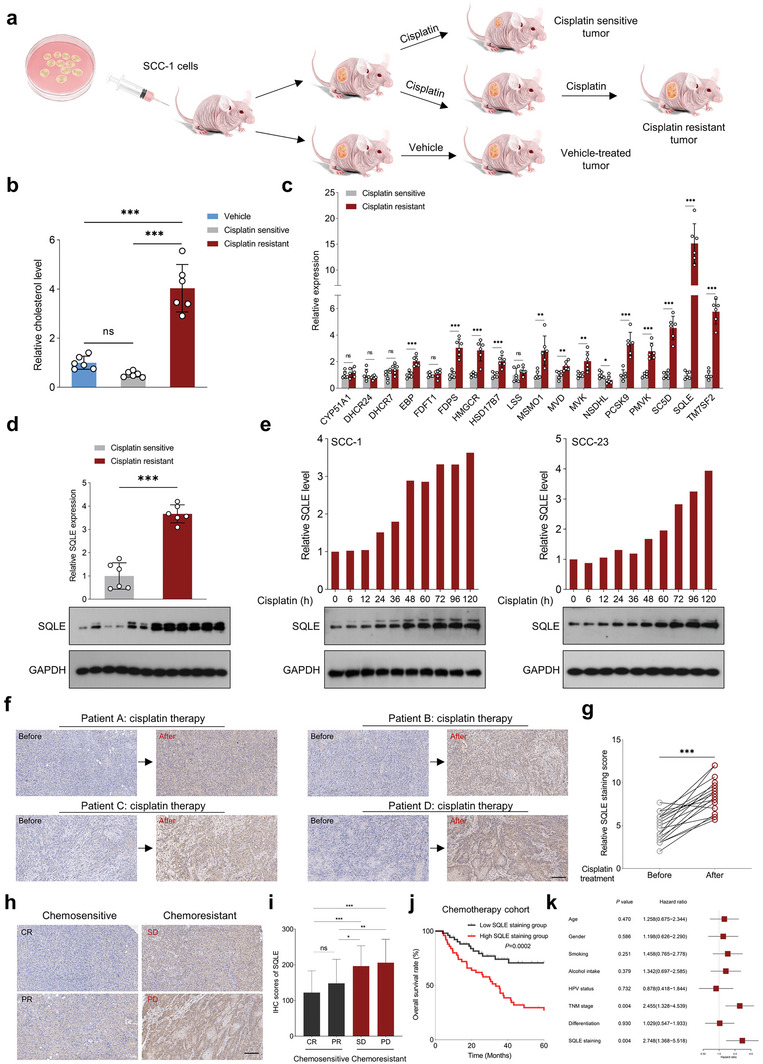
SQLE is a critical mediator of cisplatin resistance in HNSCC. a) Schematic representation of the in vivo model development for cisplatin resistance using HNSCC cell line, SCC‐1, in mice bearing HNSCC xenografts. b) Cholesterol content comparison in vehicle, cisplatin‐sensitive, and cisplatin‐resistant tumors (*n* = 6). c) Quantitative real‐time PCR (qRT‐PCR) analysis of cholesterol‐related metabolic enzyme expression in cisplatin‐sensitive and cisplatin‐resistant tumors (*n* = 6). d) Western blot analysis of SQLE expression in cisplatin‐sensitive and cisplatin‐resistant tumors (*n* = 6). e) Time‐course analysis of dynamic changes in SQLE protein expression in SCC‐1 and SCC‐23 cells treated with cisplatin (0.75 µg mL^−1^) at indicated time points. f,g) IHC staining scores of SQLE in pre‐ and post‐treatment paired tumor samples from HNSCC patients (*n* = 20) unresponsive to cisplatin‐based chemotherapy. h) Representative images of SQLE expression in tumor samples from patients with CR, PR, SD, and PD. i) Correlation between SQLE expression in clinical HNSCC specimens from an in‐house cohort before neoadjuvant chemotherapy and therapeutic efficacy (*n* = 102). j) Kaplan–Meier survival curve representing overall survival in chemotherapy‐treated basal HNSCC patients (*n* = 102) based on median SQLE expression. k) Identification of independent risk factors for predicting overall survival in HNSCC. Scale bar: 200 µm. Data were presented as mean ± standard deviation (SD). ns (not significant), *p < 0.05, **p < 0.01, ***p < 0.001 for Student's *t*‐test and one‐way ANOVA test.

To thoroughly examine the involvement of cholesterol and its related metabolic enzymes in mediating therapeutic resistance to cisplatin, we utilized SCC‐1 cell‐derived xenografts as a model to simulate acquired drug resistance in HNSCC. Tumor‐bearing mice underwent treatment with either a vehicle or cisplatin. Cisplatin‐sensitive xenografts were subsequently harvested and categorized as the cisplatin‐sensitive group. The remaining mice continued to receive cisplatin treatment until their tumors demonstrated rapid regrowth, designating them as the cisplatin‐resistant group (**Figure** **1**a). Upon measuring cholesterol levels in these xenografts, a significant enrichment was observed in cisplatin‐resistant tumors compared to both cisplatin‐sensitive tumors and vehicle‐treated xenografts (Figure 1b). These findings suggest a potential role of cholesterol in mediating cisplatin resistance in HNSCC. To further investigate this relationship, we analyzed the expression profiles of cholesterol‐related metabolic enzymes and identified *SQLE* as the most significantly upregulated gene in cisplatin‐resistant tumors compared to their cisplatin‐sensitive counterparts (Figure 1c). Additionally, elevated SQLE protein expression was observed in cisplatin‐resistant tumors (Figure 1d). SQLE levels progressively increased in response to cisplatin treatment in HNSCC cell lines, including SCC‐1 and SCC‐23 (Figure 1e), and were notably higher in cisplatin‐resistant cell lines relative to their parental counterparts (Figure S1a, Supporting Information). Supporting these experimental observations, clinical data showed that in HNSCC patients resistant to cisplatin, SQLE expression significantly increased in post‐neoadjuvant cisplatin‐based chemotherapy tumor samples compared to their matched pretreatment samples (Figure 1f,g). SQLE expression progressively increased in tumor tissues from patients with complete remission (CR), partial remission (PR), stable disease (SD), and progressive disease (PD), underscoring a significant correlation between SQLE levels and treatment efficacy (Figure 1h,i). Moreover, SQLE expression was notably elevated in cisplatin‐resistant tumors compared to cisplatin‐sensitive tumors (Figure S1b,c, Supporting Information). Taken together, these findings strongly imply that upregulated SQLE is closely linked to cisplatin resistance in HNSCC patients.

To further explore the clinical implications of SQLE in HNSCC, we evaluated *SQLE* expression across several independent HNSCC cohorts sourced from public repositories. Our extensive analysis revealed a marked upregulation of *SQLE* expression in HNSCC specimens compared to adjacent normal tissues (ANTs) or normal tissues, encompassing datasets from The Cancer Genome Atlas (TCGA) HNSCC, GSE127165, GSE37991, GSE30784, and GSE31056. Notably, in GSE30784, *SQLE* expression was significantly elevated in dysplasia tissues relative to normal tissues (Figure S2a–e, Supporting Information). Kaplan–Meier analysis demonstrated a compelling association between high *SQLE* expression and shorter overall survival/disease‐free survival time in HNSCC patients, as evidenced in TCGA HNSCC and GSE41613 cohorts (Figure S2f–h, Supporting Information). Interestingly, within GSE26549, patients with oral preneoplastic lesions and high *SQLE* expression experienced a reduced oral cancer‐free survival time compared to their counterparts with low *SQLE* expression (Figure S2i, Supporting Information). In alignment with these findings, our in‐house cohort revealed that patients exhibiting higher SQLE staining intensity faced a shorter overall survival time than those with lower intensity (Figure 1j). Furthermore, late‐stage tumor samples displayed significantly elevated SQLE expression compared to early‐stage samples (Figure S2j, Supporting Information). Importantly, both univariate and multivariate analyses substantiated the notion that SQLE upregulation in tumor specimens serves as an independent risk factor for HNSCC patients (Figure 1k; Figure S2k, Supporting Information). Considering the unique oncogenic drivers of HPV‐positive and HPV‐negative HNSCC subtypes, we further dissected our in‐house HNSCC cohort for a more refined analysis. Our results demonstrated a marked increase in SQLE staining intensity in cisplatin‐treated tumor samples compared to pretreated samples, irrespective of HPV status (Figure S3a,b, Supporting Information). Moreover, patients with high SQLE staining exhibited poorer overall survival than those with low staining, regardless of their HPV status (Figure S3c,d, Supporting Information). For the HPV‐negative group, we observed a progressive increase in SQLE expression across tumor samples with varying therapeutic responses (Figure S3e, Supporting Information). Importantly, multivariate analysis established that SQLE upregulation served as an independent risk factor for this group (Figure S3f, Supporting Information). Conversely, for the HPV‐positive group, no significant difference in SQLE expression was detected among tumor tissues with differing therapeutic responses (Figure S3g, Supporting Information). Furthermore, multivariate analysis indicated that SQLE was not an independent risk factor for this group (Figure S3h, Supporting Information).

### 2.2 Cisplatin Exposure Induces SQLE Transcriptional Activation by the *β*‐Catenin‐TCF4 Complex in HNSCC Cells

To elucidate the mechanism underlying cisplatin‐induced SQLE expression in HNSCC cells, we initially assessed *SQLE* mRNA expression following cisplatin treatment. Our observations revealed a marked increase in *SQLE* expression upon cisplatin exposure (Figure S4a, Supporting Information), suggesting that transcriptional regulation may facilitate SQLE expression. Employing bioinformatic tools such as TFBSTools, JASPAR2016, and Biostrings, we identified TCF4 as a potential transcription factor binding to the SQLE promoter (GGCACCTGCT). The TCF4 binding motif sequence logo from the JASPAR database was illustrated in Figure S4b of the Supporting Information. Given the pivotal role of the *β*‐catenin/TCF4 complex in promoting tumorigenesis and mediating drug resistance,^[^ ^19^, ^20^ ^]^ we hypothesized that *β*‐catenin/TCF4 functions as an upstream regulator of SQLE. Western blot and qRT‐PCR assays corroborated that depleting *β*‐catenin or TCF4 substantially diminished SQLE expression in HNSCC cells, while their overexpression resulted in increased SQLE expression (Figure S4c–f, Supporting Information). Moreover, our luciferase reporter assay indicated a significant decrease in relative luciferase activities in cells with *β*‐catenin or TCF4 depletion, and an enhancement in activities when *β*‐catenin or TCF4 were overexpressed (Figure S4g–j, Supporting Information). This evidence suggests that the *β*‐catenin/TCF4 complex transcriptionally modulates SQLE expression in HNSCC cells. Chromatin immunoprecipitation (ChIP)‐qPCR analysis further detected TCF4 enrichment in the SQLE promoter region (Figure S4k, Supporting Information). Notably, our data also demonstrated a progressive elevation in *β*‐catenin expression in HNSCC cells upon cisplatin exposure (Figure S4l, Supporting Information). These findings propose that the cisplatin‐induced upregulation of the *β*‐catenin/TCF4 signaling pathway subsequently results in increased SQLE expression in HNSCC cells.

### 2.3 SQLE Depletion Potentiates Cisplatin Sensitivity in Resistant HNSCC Cells Both In Vitro and In Vivo

**Figure 2:**
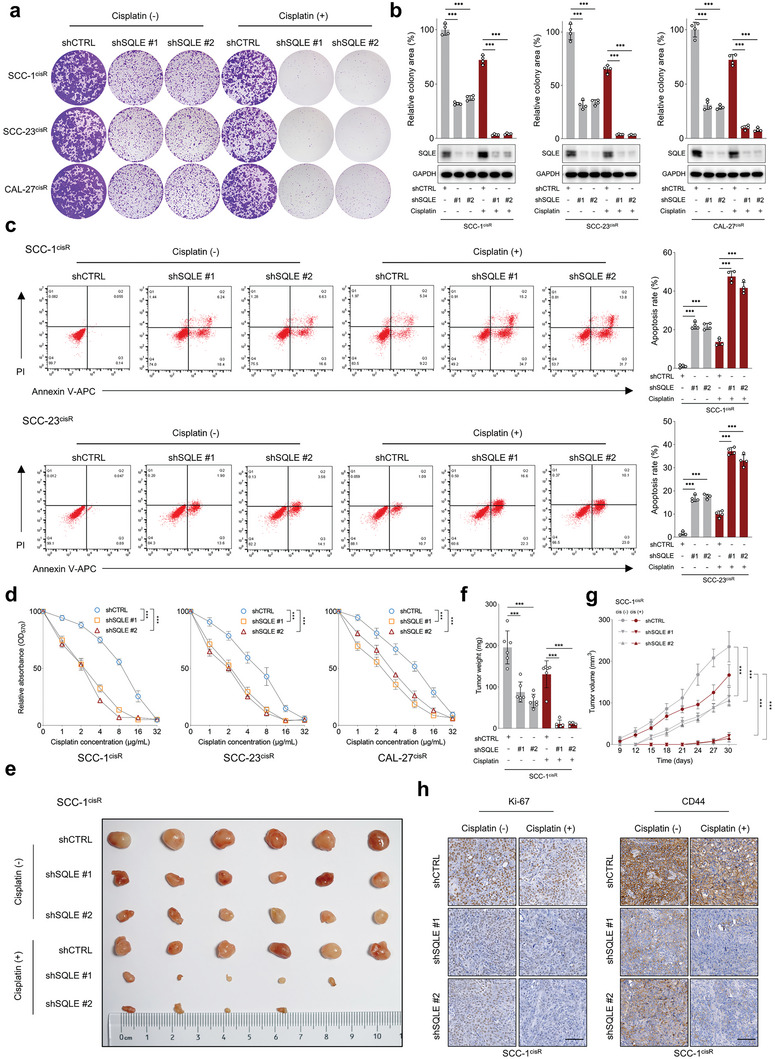
SQLE depletion restores cisplatin sensitivity in cisplatin‐resistant HNSCC cells both in vitro and in vivo. a,b) Colony formation potential of SCC‐1^cisR^, SCC‐23^cisR^, and CAL‐27^cisR^ cells with SQLE depletion, with or without cisplatin treatment (*n* = 4). Cisplatin concentrations: SCC‐1^cisR^, 8 µg mL^−1^; SCC‐23^cisR^, 5 µg mL^−1^; CAL‐27^cisR^, 8 µg mL^−1^. SQLE knockdown efficiency determined by western blotting. c) Apoptosis rate of SCC‐1^cisR^ and SCC‐23^cisR^ cells with SQLE depletion in the presence or absence of cisplatin (*n* = 4). d) Cell viability of SCC‐1^cisR^, SCC‐23^cisR^, and CAL‐27^cisR^ cells with SQLE depletion following dose‐gradient cisplatin exposure (*n* = 4). e–g) Effect of SQLE depletion on tumor growth in SCC‐1^cisR^ cells with or without cisplatin treatment. Mice were treated with vehicle control or cisplatin (5 mg kg^−1^ i.p. twice per week) starting 1 week after tumor cell inoculation (*n* = 6). Tumor volume and animal health status were monitored. h) IHC scores of Ki‐67 and CD44 in xenograft tumor tissues with SQLE knockdown, in the presence or absence of cisplatin (*n* = 6). Scale bar: 100 µm. Data were presented as mean ± SD. ***p < 0.001 for one‐way ANOVA test.

To probe the influence of SQLE on the malignancy of HNSCC cells in the presence or absence of cisplatin, we employed lentivirus‐mediated SQLE shRNA to specifically knock down SQLE expression in SCC‐1^cisR^, SCC‐23^cisR^, and CAL‐27^cisR^ cells. Remarkably, SQLE knockdown diminished the colony‐forming potential of HNSCC cells in the absence of cisplatin, and the combination of SQLE depletion and cisplatin treatment exhibited a substantially greater inhibitory effect on colony formation than cisplatin alone (**Figure** **2**a,b). In tandem, the concurrent application of shSQLE and cisplatin notably enhanced apoptosis induction in SCC‐1^cisR^ and SCC‐23^cisR^ cells (Figure 2c). Furthermore, MTT assay results revealed that SQLE depletion sensitized cancer cells to cisplatin treatment (Figure 2d). Employing a xenograft mouse model with SCC‐1^cisR^ or SCC‐23^cisR^ cells with or without SQLE depletion, we investigated SQLE's impact on tumor growth. Our findings demonstrated that SQLE depletion in SCC‐1^cisR^ cells led to a reduction in tumor size, weight, and volume, while the concurrent administration of cisplatin significantly amplified the suppressive effects of SQLE depletion on tumor growth (Figure 2e–g). Additionally, we observed a marked decrease in Ki‐67 and CD44 staining intensities in xenograft tumor tissues originating from cancer cells with SQLE depletion, as opposed to control cells. Importantly, the combination of shSQLE and cisplatin yielded a more pronounced attenuation of Ki‐67 and CD44 expression than using shSQLE or cisplatin individually (Figure 2h; Figure S5a, Supporting Information). Analogous results were identified in SCC‐23^cisR^ cells (Figure S5b–f, Supporting Information). Collectively, these findings underscore the critical role of SQLE expression in tumor growth and cisplatin resistance in HNSCC, while also revealing that targeting SQLE can sensitize cisplatin‐resistant HNSCC cells to cisplatin.

It has been reported that SQLE inhibition hampers homologous recombination repair,^[^ ^21^ ^]^ a critical pathway in repairing cisplatin‐induced DNA damage and mediating drug resistance. We investigated the expression of DNA damage repair‐related proteins in SCC‐1^cisR^ and SCC‐23^cisR^ cells following SQLE depletion. Western blot analysis revealed that SQLE depletion exerted minimal effects on the expression of p‐ATM, p‐CHK2, and *γ*‐H2AX (Figure S6, Supporting Information). This finding suggests that the sensitization of SCC‐1^cisR^ and SCC‐23^cisR^ cells to cisplatin due to SQLE inhibition might not be attributed to impaired DNA damage repair.

### 2.4 SQLE Regulates Cholesterol Levels to Preserve Cancer Stemness and Tumorigenic Characteristics

**Figure 3:**
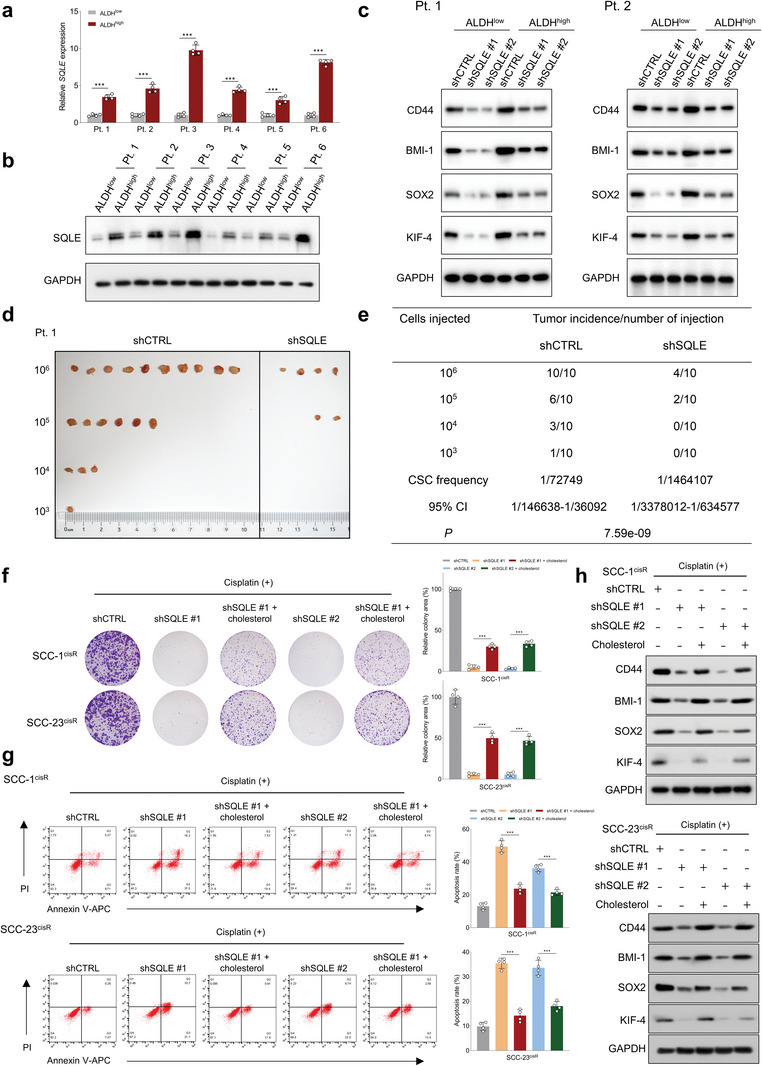
SQLE promotes tumorigenic phenotypes and cancer stemness in HNSCC cells through modulating cholesterol homeostasis. a,b) qRT‐PCR and western blot analyses of SQLE mRNA and protein expression in ALDH^high^ and ALDH^low^ cells isolated from six HNSCC patients (*n* = 4). c) Western blot analysis of the impact of SQLE depletion on cancer stem cell‐related marker expression (CD44, BMI‐1, SOX2, and KIF‐4) in ALDH^high^ and ALDH^low^ primary HNSCC cells from patient case #1 (Pt. 1) and case #2 (Pt. 2). d,e) In vivo limiting dilution analysis of EpCAM^+^ALDH^high^ tumor cells from Pt. 1 infected with shCTRL and shSQLE lentiviruses (*n* = 10). Frequency of tumor‐initiating cells at each injected cell dose is shown. Differences in CSC frequencies calculated using the Extreme Limiting Dilution Analysis (ELDA) software. f,g) Effects of cholesterol repletion (20 µg mL^−1^) on colony formation potential and cellular apoptosis in SCC‐1^cisR^ and SCC‐23^cisR^ cells with SQLE depletion in the presence of cisplatin (*n* = 4). h) Effects of exogenous cholesterol (20 µg mL^−1^) on expression of CD44, BMI‐1, SOX2, and KIF‐4 in SCC‐1^cisR^ and SCC‐23^cisR^ cells with SQLE depletion and cisplatin treatment. Data were presented as mean ± SD. ***p < 0.001 for Student's *t*‐test and one‐way ANOVA test.

It is well‐established that cancer stem cells (CSCs) play a significant role in cisplatin resistance, and increased aldehyde dehydrogenase (ALDH) activity has been observed in CSCs derived from both human primary HNSCC tissues and cell lines.^[^ ^22^, ^23^, ^24^ ^]^ Thus, we first evaluated the effects of SQLE depletion on the cancer stemness in HNSCC using ALDEFLUOR assay and sphere formation assay. Our data demonstrated that SQLE knockdown decreased the proportion of ALDH‐positive cells and the sphere‐forming capacity of SCC‐1^cisR^ and SCC‐23^cisR^ cells, while cisplatin administration significantly enhanced the suppressive effects of shSQLE (Figure S7a‐c, Supporting Information). We then sorted ALDH^low^ cancer cells and ALDH^high^ CSC cells in tumors obtained from six HNSCC patients. As shown in **Figure** **3**a,b, both SQLE mRNA and protein expression were consistently higher in ALDH^high^ CSC cells compared to ALDH^low^ cancer cells. Western blot analysis revealed that SQLE depletion substantially reduced the expression of cancer stemness‐related proteins, including CD44, BMI1, SOX2, and KIF‐4, in ALDH^high^ primary HNSCC cells. Notably, SQLE depletion also remarkably inhibited the expression of these proteins in ALDH^low^ primary HNSCC cells, despite their relatively low expression levels (Figure 3c). Utilizing the limiting dilution tumor initiation assay, we further examined SQLE's role in regulating the tumorigenic potential of CSCs, uncovering that SQLE depletion dramatically hindered the tumor‐initiating capacity of ALDH^high^ CSCs in vivo (Figure 3d,e). Conversely, ectopic SQLE expression increased the tumorigenic potential of ALDH^high^ CSCs in nude mice (Figure S8a,b, Supporting Information).

While the connection between cholesterol content and HNSCC cancer stemness is not yet fully understood, increased cholesterol biosynthesis has been reported as crucial for augmenting CSCs in breast and liver cancer.^[^ ^25^, ^26^ ^]^ We initially evaluated whether SQLE was a pivotal metabolic enzyme for maintaining cholesterol homeostasis in HNSCC cells or tissues. Our findings indicated that SQLE depletion significantly lowered cholesterol levels in SCC‐1^cisR^ or SCC‐23^cisR^ cells (Figure S9a, Supporting Information). Furthermore, we classified HNSCC patients into *SQLE*‐high and *SQLE*‐low expression groups based on median *SQLE* expression in multiple independent HNSCC cohorts, and identified an enrichment of the cholesterol homeostasis gene signature in the *SQLE*‐high expression group (Figure S9b, Supporting Information). These results suggest that SQLE is essential for maintaining cholesterol homeostasis in HNSCC. We hypothesized that SQLE might foster tumorigenic properties and cancer stemness in HNSCC cells through cholesterol level modulation. Our experiments showed that the combination of SQLE depletion and cisplatin nearly abolished the colony‐forming capacity of SCC‐1^cisR^ and SCC‐23^cisR^ cells, while the addition of cholesterol partially restored these suppressive effects (Figure 3f). Likewise, the combined treatment of shSQLE and cisplatin significantly increased the apoptotic rate of SCC‐1^cisR^ and SCC‐23^cisR^ cells, and the introduction of cholesterol partially counteracted the apoptosis‐inducing effects (Figure 3g). Importantly, the exogenous cholesterol also partially restored the expression of CD44, BMI1, SOX2, and KIF‐4, which had been suppressed by shSQLE and cisplatin in SCC‐1^cisR^ and SCC‐23^cisR^ cells (Figure 3h).

### 2.5 SQLE Post‐Transcriptionally Regulates c‐Myc Expression

**Figure 4:**
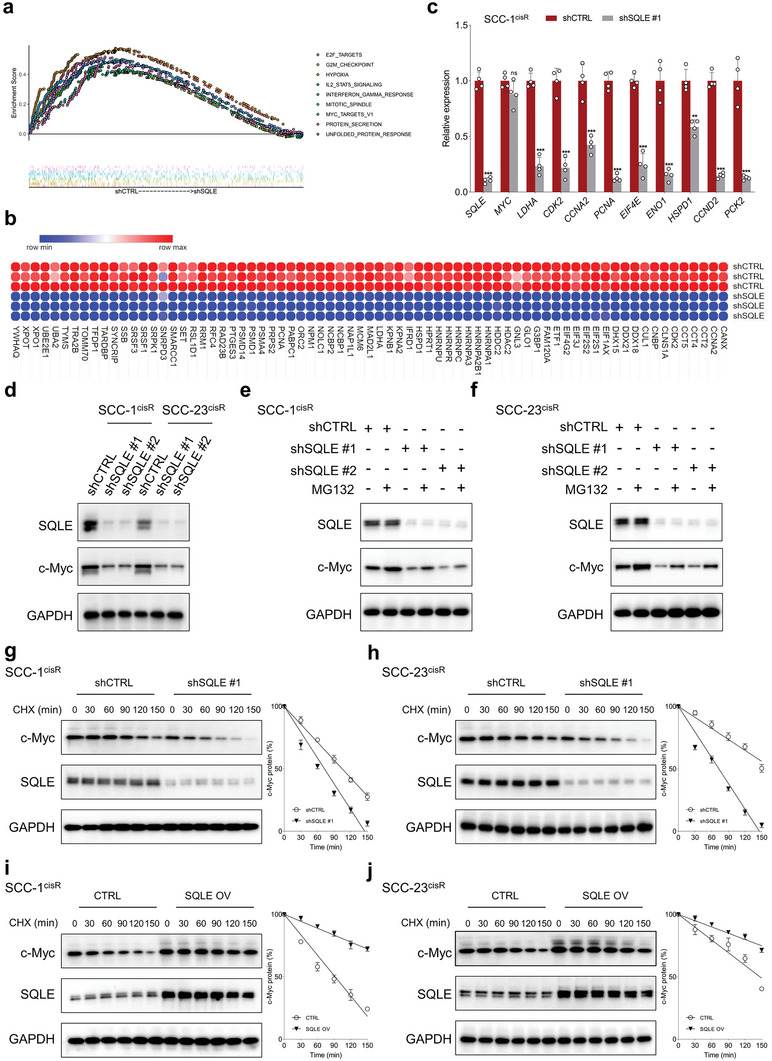
SQLE inhibition destabilizes c‐Myc and impairs its transcriptional activity. a) GSEA analysis shows significant enrichment of genes in the MYC_TARGETS_V1 molecular signature in SCC‐23^cisR^ cells without SQLE depletion. b) Heatmap depicting aberrant expression of c‐Myc target genes upon SQLE depletion in SCC‐23^cisR^ cells. c) qRT‐PCR analysis of representative c‐Myc‐induced target genes in SCC‐1^cisR^ cells following SQLE depletion (*n* = 4). d) Expression of c‐Myc and SQLE in SCC‐1^cisR^ and SCC‐23^cisR^ cells upon SQLE knockdown. e,f) SQLE‐depleted SCC‐1^cisR^ and SCC‐23^cisR^ cells treated with MG132 (20 µm, 8 h) before harvest, with SQLE and c‐Myc expression analyzed by western blotting. g,h) Time‐course analysis of c‐Myc and SQLE expression in SCC‐1^cisR^ and SCC‐23^cisR^ cells with or without SQLE depletion. Cells treated with CHX (100 µg mL^−1^) for varying periods. i,j) SCC‐1^cisR^ or SCC‐23^cisR^ cells with or without SQLE overexpression were treated with 100 µg mL^−1^ CHX for indicated time periods; western blotting performed to analyze dynamic changes in c‐Myc and SQLE. Data were presented as mean ± SD. ns (not significant), **p < 0.01, ***p < 0.001 for Student's *t*‐test.

To unravel the underlying mechanisms by which the SQLE‐cholesterol axis influences the tumorigenesis of HNSCC, we performed an unbiased, genome‐wide transcriptomic analysis of SQLE‐depleted SCC‐23^cisR^ cells utilizing RNA sequencing. Gene set enrichment analysis (GSEA) analysis demonstrated that MYC_TARGETS_V1 (NES = 1.374, FDR = 0.033) was among the most enriched signatures (ranking only after E2F_TARGETS, G2M_CHECKPOINT, and PROTEIN_SECRETION) in the shCTRL group compared to the shSQLE group (**Figure** **4**a; Figure S10a, Supporting Information). The enrichment of E2F_TARGETS and G2M_CHECKPOINT signatures in the shCTRL group indicates that SQLE critically contributes to regulating the proliferation capacity of HNSCC cells, consistent with our findings in Figure 2a. Furthermore, we analyzed independent HNSCC cohorts from publicly available datasets. Dividing the HNSCC cohort into *SQLE*‐high and ‐low expression groups based on median *SQLE* expression, GSEA results revealed that MYC_TARGETS_V1 and/or MYC_TARGETS_V2 were enriched in the *SQLE*‐high expression group across multiple HNSCC cohorts, including GSE25099, GSE25727, GSE26549, GSE27020, GSE31056, GSE37991, GSE41613, and GSE85446 (Figure S10b,c, Supporting Information). This supplementary evidence reinforces our RNA‐seq data, suggesting that SQLE might impact the expression of c‐Myc targets. Detailed alterations in c‐Myc downstream target expression following SQLE depletion are depicted in Figure 4b. We posited that SQLE inactivation would affect the global c‐Myc transcriptional program. Supporting this hypothesis, qRT‐PCR validation confirmed that SQLE depletion led to significant downregulation of c‐Myc‐targeted genes (Figure 4c), underscoring SQLE's pivotal role in sustaining c‐Myc transcriptional activity.

To investigate the mechanisms underlying SQLE's regulation of c‐Myc, we assessed c‐Myc mRNA and protein expression following SQLE depletion. SQLE inactivation did not markedly decrease c‐Myc mRNA levels in SCC‐1^cisR^ cells (Figure 4c). However, SQLE depletion substantially inhibited c‐Myc protein expression in both SCC‐1^cisR^ and SCC‐23^cisR^ cells (Figure 4d), suggesting that SQLE modulates c‐Myc expression through a post‐transcriptional mechanism. Notably, the proteasome inhibitor MG132 effectively rescued the prominent decline in c‐Myc protein abundance in both SCC‐1^cisR^ and SCC‐23^cisR^ cells (Figure 4e,f). Treating SCC‐1^cisR^ and SCC‐23^cisR^ cells with protein synthesis inhibitor cycloheximide (CHX) showed that SQLE depletion diminished c‐Myc expression by shortening c‐Myc's half‐life (Figure 4g,h). By contrast, SQLE overexpression significantly enhanced c‐Myc expression by decreasing the degradation rate of c‐Myc protein (Figure 4i,j). Collectively, these findings reveal that SQLE augments c‐Myc stability by impeding its proteasomal degradation.

### 2.6 SQLE Orchestrates c‐Myc Stability via Regulation of Lipid‐Raft‐Associated Akt Activity

**Figure 5:**
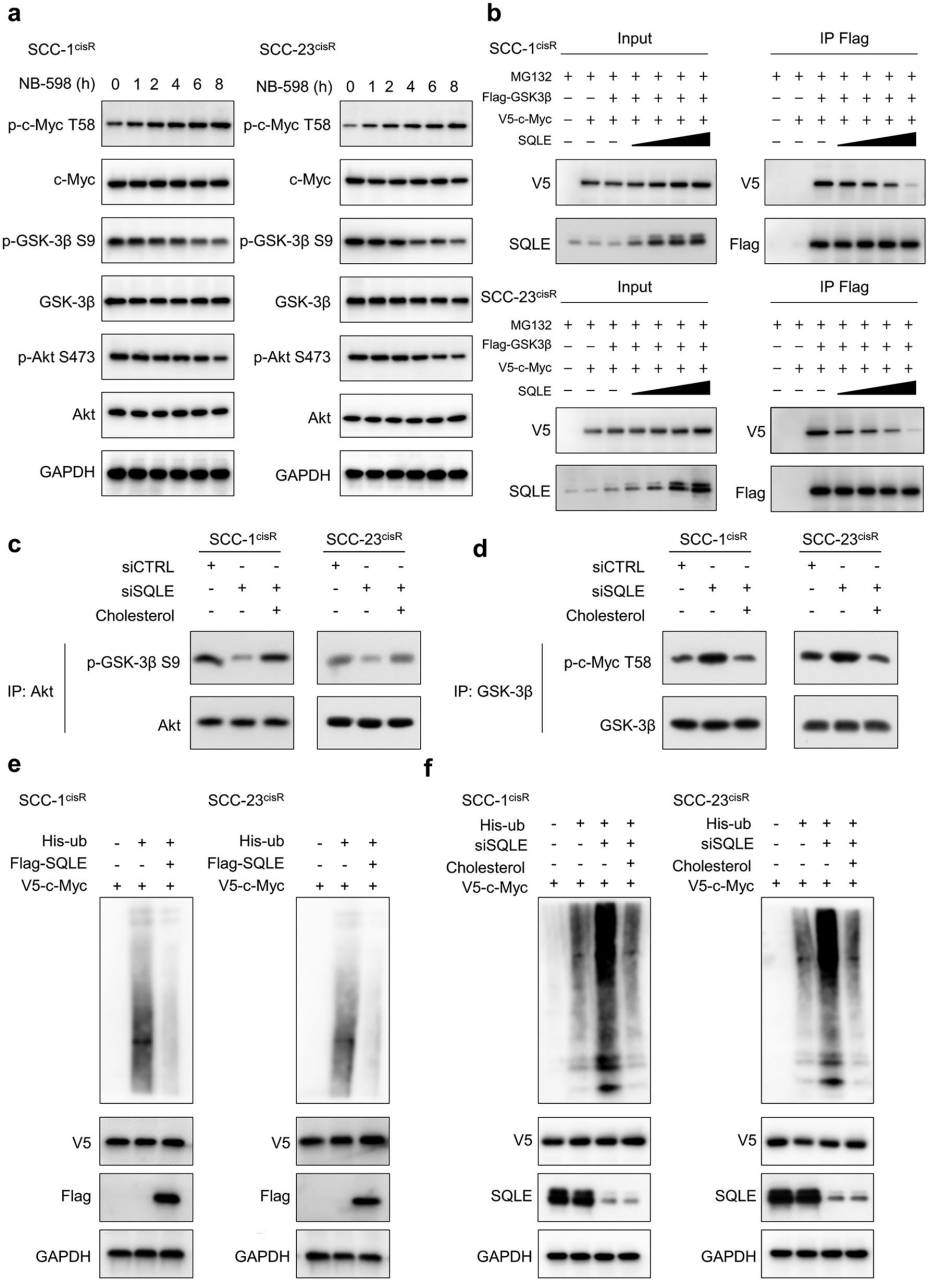
SQLE promotes c‐Myc stabilization by regulating phosphorylation of c‐Myc at T58. a) Time‐course analysis of p‐c‐Myc T58, c‐Myc, p‐GSK‐3*β* S9, GSK‐3*β*, p‐Akt S473, and Akt expression in SCC‐1^cisR^ and SCC‐23^cisR^ cells treated with NB‐598 (1 µm). b) Protein interaction between GSK‐3*β* and c‐Myc in SCC‐1^cisR^ and SCC‐23^cisR^ cells with indicated modifications. Flag‐GSK3*β* (1 mg), V5‐c‐Myc (1 mg), and/or increasing doses of SQLE (1, 2, and 4 mg) were transfected into SCC‐1^cisR^ and SCC‐23^cisR^ cells for 48 h as indicated. Cell lysates were harvested and subjected to Co‐IP and western blotting. c) SCC‐1^cisR^ and SCC‐23^cisR^ cells transfected with siCTRL or siSQLE were incubated in medium with or without 20 µg mL^−1^ cholesterol for 24 h. GSK3*β* fusion protein was used as Akt substrate, and Co‐IP was performed with anti‐Akt antibody. Expression of p‐GSK‐3*β* S9 analyzed using western blotting. d) SCC‐1^cisR^ and SCC‐23^cisR^ cells with or without SQLE depletion were incubated in medium with or without 20 µg mL^−1^ cholesterol for 24 h. c‐Myc fusion protein was used as GSK‐3*β* substrate, and Co‐IP was performed with anti‐GSK‐3*β* antibody. Expression of p‐c‐Myc T58 determined using western blotting. e,f) His‐tagged ubiquitin and V5‐c‐Myc were cotransfected with Flag‐SQLE or siSQLE into SCC‐1^cisR^ and SCC‐23^cisR^ cells in the absence or presence of cholesterol. Indicated cell lysates were harvested and subjected to Co‐IP.

c‐Myc protein levels and activity are stringently controlled by the ubiquitin‐proteasome system, which requires GSK3*β*‐mediated phosphorylation of c‐Myc at T58. We evaluated whether SQLE inhibition impacted the expression of p‐c‐Myc T58.^[^ ^27^ ^]^ Time‐course expression analysis demonstrated that treatment with the SQLE inhibitor NB‐598 in both SCC‐1^cisR^ and SCC‐23^cisR^ cells led to a significant increase in p‐c‐Myc T58 (**Figure** **5**a). Concurrently, the expression of the inactive form of GSK‐3*β* (p‐GSK‐3*β* S9) decreased following the addition of NB‐598 (Figure 5a). It is well‐established that GSK‐3*β* at Ser9 is phosphorylated by Akt.^[^ ^27^ ^]^ We subsequently assessed the upstream of phosphorylated GSK‐3*β* by examining the time course of changes in the level of phosphorylated Akt at S473 (p‐Akt S473). Results revealed a progressive reduction in p‐Akt S473 expression following SQLE inhibition (Figure 5a). Previous study has suggested that SQLE modulates AKT activity through a PTEN‐dependent mechanism.^[^ ^26^ ^]^ Our findings revealed that PTEN levels were markedly diminished in HNSCC cell lines, particularly in SCC‐1^cisR^, SCC‐23^cisR^, and HN30, compared to normal epithelial cells (NHOK, NHEK) (Figure S11a, Supporting Information). Moreover, neither SQLE depletion nor overexpression appeared to significantly impact PTEN expression (Figure S11b,c, Supporting Information), implying that SQLE might not influence AKT activities via a PTEN‐dependent mechanism in SCC‐1^cisR^ and SCC‐23^cisR^ cells. Furthermore, increasing SQLE expression abrogated the interaction between c‐Myc and GSK‐3*β* in both SCC‐1^cisR^ and SCC‐23^cisR^ cells (Figure 5b). We hypothesized that SQLE might regulate the Akt/GSK‐3*β*/c‐Myc pathway by modulating cholesterol levels. To determine whether the effect of SQLE depletion on Akt activity was cholesterol‐dependent, exogenous cholesterol complexes were added to cells simultaneously with siSQLE. As depicted in Figure 5c, SQLE depletion diminished the binding of Akt to p‐GSK‐3*β* S9, and cholesterol repletion counteracted the suppressive effects of SQLE depletion on Akt activity, suggesting this effect was mediated by a reduction in membrane cholesterol. Similarly, treatment with siSQLE promoted the interaction between GSK‐3*β* and p‐c‐Myc T58, while the addition of cholesterol reversed this enhancing effect (Figure 5d). We next examined the regulation of c‐Myc ubiquitination by SQLE. Overexpression of SQLE reduced c‐Myc polyubiquitination in both SCC‐1^cisR^ and SCC‐23^cisR^ cells (Figure 5e). Conversely, SQLE depletion increased c‐Myc polyubiquitylation, and cholesterol repletion mitigated the enhancing effect of SQLE depletion on c‐Myc polyubiquitylation (Figure 5f). Collectively, these data demonstrate that SQLE stabilizes c‐Myc expression by activating the Akt/GSK‐3*β*/c‐Myc pathway, and this signaling cascade is cholesterol‐dependent.

**Figure 6:**
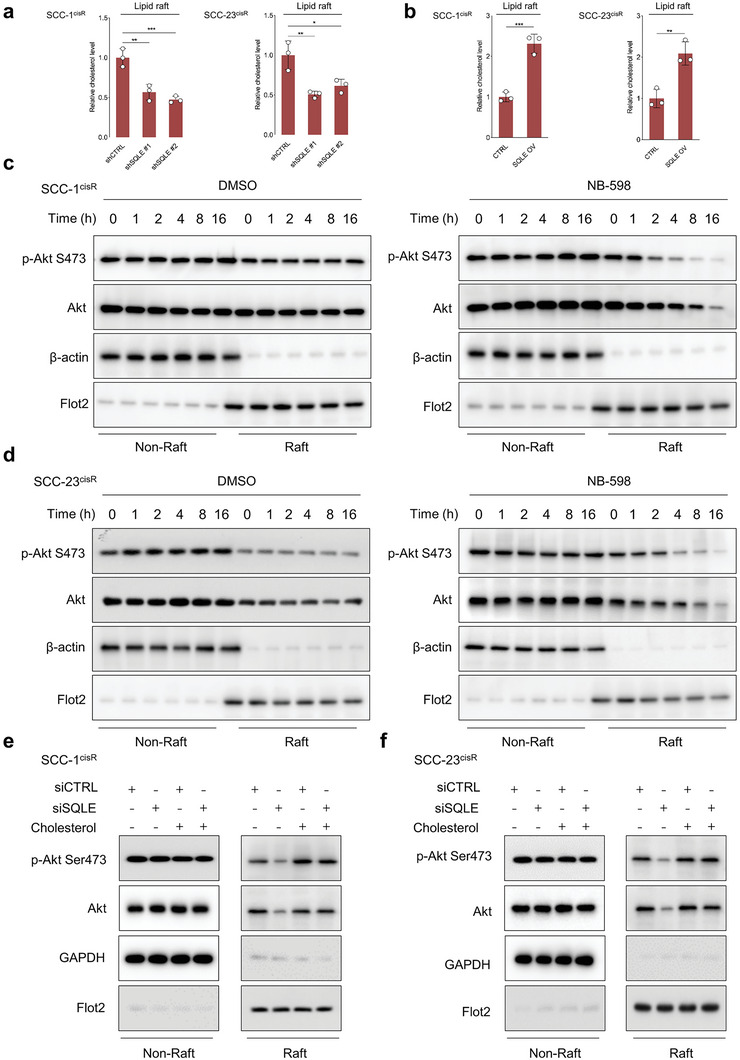
SQLE depletion reduces cholesterol content in lipid rafts of HNSCC cells and suppresses phosphorylation of raft‐associated Akt. a,b) Relative cholesterol content in lipid rafts from cells with indicated treatments (*n* = 3). c,d) Time‐course analysis of p‐Akt S473 and Akt expression in lipid raft and nonraft components of SCC‐1^cisR^ and SCC‐23^cisR^ cells treated with DMSO or NB‐598 (1 µm). Western blot analyses performed with indicated antibodies. *β*‐actin or Flot2 serves as internal control for determining protein expression in nonraft components or lipid rafts, respectively. e,f) Expression of p‐Akt S473 and Akt in lipid raft and nonraft components of SQLE‐depleted cells or CTRL cells with or without addition of exogenous cholesterol. Data were presented as mean ± SD. *p < 0.05, **p < 0.01, ***p < 0.001 for Student's *t*‐test and one‐way ANOVA test.

Lipid rafts are membrane microdomains enriched with cholesterol, glycosphingolipids, and receptor proteins, serving as platforms for various critical signaling pathways.^[^ ^28^ ^]^ To determine whether SQLE depletion or overexpression alters the cholesterol composition of lipid raft microdomains, we isolated lipid rafts using discontinuous sucrose density gradient ultracentrifugation. Western blotting analysis indicated that lipid rafts were primarily located in fractions 5–7 (Figure S12, Supporting Information). Our results demonstrated that SQLE depletion significantly reduced cholesterol content in lipid rafts, while SQLE overexpression substantially increased cholesterol content within these domains (**Figure** **6**a,b). These findings provide evidence that SQLE is crucial for maintaining cholesterol homeostasis in membrane rafts. Considering that SQLE significantly impacts Akt activity in whole cell lysates and cholesterol content in lipid rafts, we next investigated the effect of SQLE inhibition on lipid raft‐associated Akt activity. Our results showed that treatment with NB‐598 had minimal impact on Akt localization and the expression of p‐Akt S473 in nonraft components. By contrast, time‐course analysis revealed that the expression of raft‐localized Akt and raft‐localized p‐Akt S473 progressively decreased in NB‐598 treated cells, while no such decrease was observed in DMSO‐treated cells (Figure 6c,d). More importantly, as demonstrated in Figure 6e,f, SQLE depletion suppressed the expression of raft‐localized Akt and raft‐localized p‐Akt S473, and cholesterol repletion restored their expression in rafts.

We further investigated the effects of SQLE depletion on c‐Myc stability in human stem cells, employing human dental pulp stem cells (hDPSCs) and human periodontal ligament stem cells (hPDLSCs) as experimental systems. Our results revealed that SQLE depletion or overexpression exerted minimal influence on c‐Myc protein and mRNA expression in both hDPSCs and hPDLSCs (Figure S13a–d, Supporting Information). The CHX chase assay substantiated that the degradation rate of c‐Myc remained largely unchanged in both SQLE‐depleted and control cells (Figure S13e,f, Supporting Information). Moreover, the western blot assay detected no significant alterations in the expression of raft‐localized Akt following SQLE depletion (Figure S13g,h, Supporting Information). These findings support the notion that SQLE functions distinctly in cancer cells compared to normal cells.

### 2.7 C‐Myc as a Functional Downstream Target of SQLE in HNSCC

Upon establishing the crucial mechanisms by which SQLE modulates c‐Myc stability and expression, we proceeded to investigate if c‐Myc serves as a functional downstream target of SQLE. c‐Myc expression is markedly elevated in cisplatin‐resistant cell lines compared to their cisplatin‐sensitive counterparts (Figure S14, Supporting Information). We then examined whether the impact of SQLE depletion could be emulated by c‐Myc depletion. The combination of c‐Myc knockdown and cisplatin treatment led to a markedly enhanced inhibitory effect on colony formation and augmented apoptosis induction in SCC‐1^cisR^ and SCC‐23^cisR^ cells compared to cisplatin treatment alone (Figure S15a–c, Supporting Information). Moreover, c‐Myc depletion sensitized cancer cells to cisplatin, as evidenced by the MTT assay (Figure S15d, Supporting Information). Notably, c‐Myc plays a critical role in maintaining cancer cell stemness. c‐Myc mRNA and protein expression levels were consistently elevated in ALDH^high^ CSC cells relative to ALDH^low^ cancer cells (Figure S15e,f, Supporting Information). c‐Myc depletion significantly diminished the expression of CD44, BMI1, SOX2, and KIF‐4 in both ALDH^high^ and ALDH^low^ primary HNSCC cells (Figure S15g, Supporting Information).

**Figure 7:**
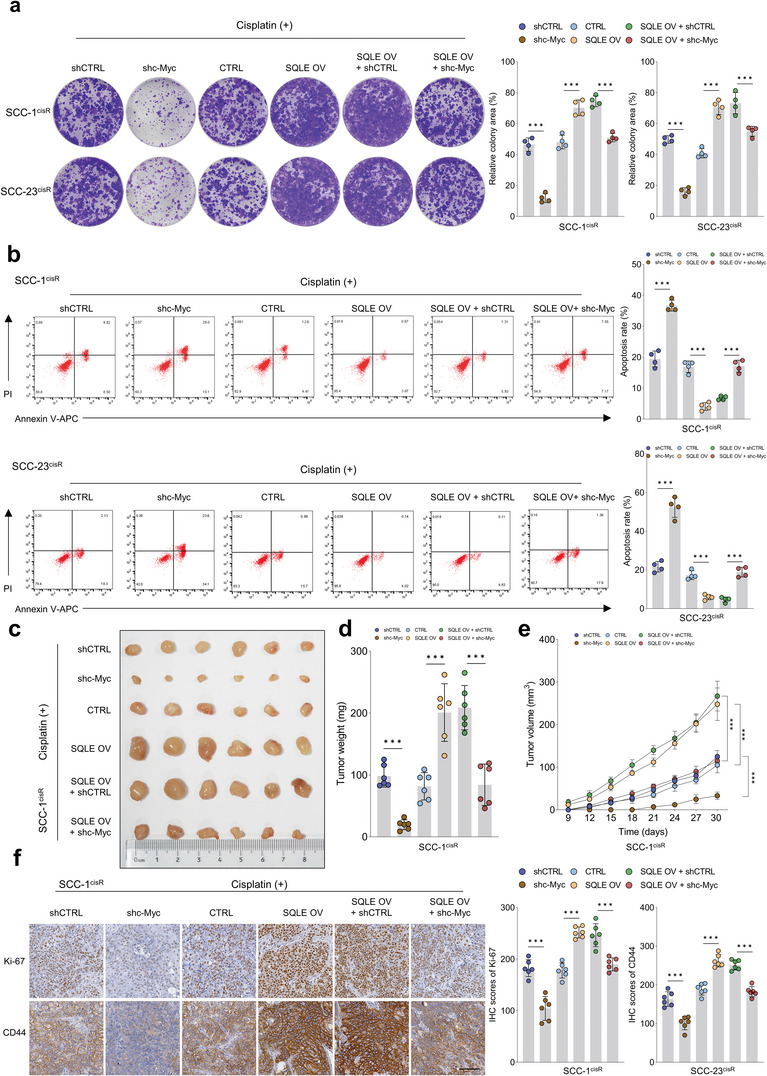
c‐Myc is a functional downstream target of SQLE. a) Colony formation potential of SCC‐1^cisR^ and SCC‐23^cisR^ cells with indicated modifications (*n* = 4). b) Apoptosis rate of SCC‐1^cisR^ and SCC‐23^cisR^ cells with indicated treatments (*n* = 4). c–e) Effects of c‐Myc depletion on tumor size, weight, and volume of SQLE‐overexpressing SCC‐1^cisR^ cells (*n* = 6). f) IHC scores of Ki‐67 and CD44 in xenograft tumor tissues with indicated treatments (*n* = 6). Scale bar: 100 µm. Data were presented as mean ± SD. ***p < 0.001 for one‐way ANOVA test.

The colony formation assay demonstrated that c‐Myc depletion counteracted the enhanced colony‐forming capacity of SCC‐1^cisR^ and SCC‐23^cisR^ cells, which was induced by SQLE overexpression in the presence of cisplatin (**Figure** **7**a). Likewise, enforced SQLE expression reduced the apoptosis rate of SCC‐1^cisR^ and SCC‐23^cisR^ cells exposed to cisplatin, while c‐Myc knockdown abrogated this suppressive effect (Figure 7b). The subcutaneous tumor model of SCC‐1^cisR^ cells further illustrated that tumor size, weight, and volume were significantly increased in the SQLE‐overexpressing group compared to the control group when treated with cisplatin, indicating that SQLE upregulation enhances cisplatin resistance in nude mice. Importantly, c‐Myc depletion nearly eradicated the tumor‐promoting effects of SQLE overexpression in the presence of cisplatin (Figure 7c–e). Furthermore, Ki‐67 and CD44 expression were substantially elevated in xenograft tissues from the SQLE‐overexpressing group, and c‐Myc depletion mitigated these enhancements (Figure 7f). Comparable findings were observed in SCC‐23^cisR^ cells (Figure S16a–d, Supporting Information). c‐Myc depletion dramatically impeded the tumor‐initiating capacity of SQLE‐overexpressing SCC‐1 cells (Figure S17a–c, Supporting Information). Taken together, these results provide compelling evidence that SQLE promotes HNSCC tumorigenesis by regulating c‐Myc expression.

### 2.8 SQLE Plays a Pivotal Role in HNSCC Initiation and Progression

**Figure 8:**
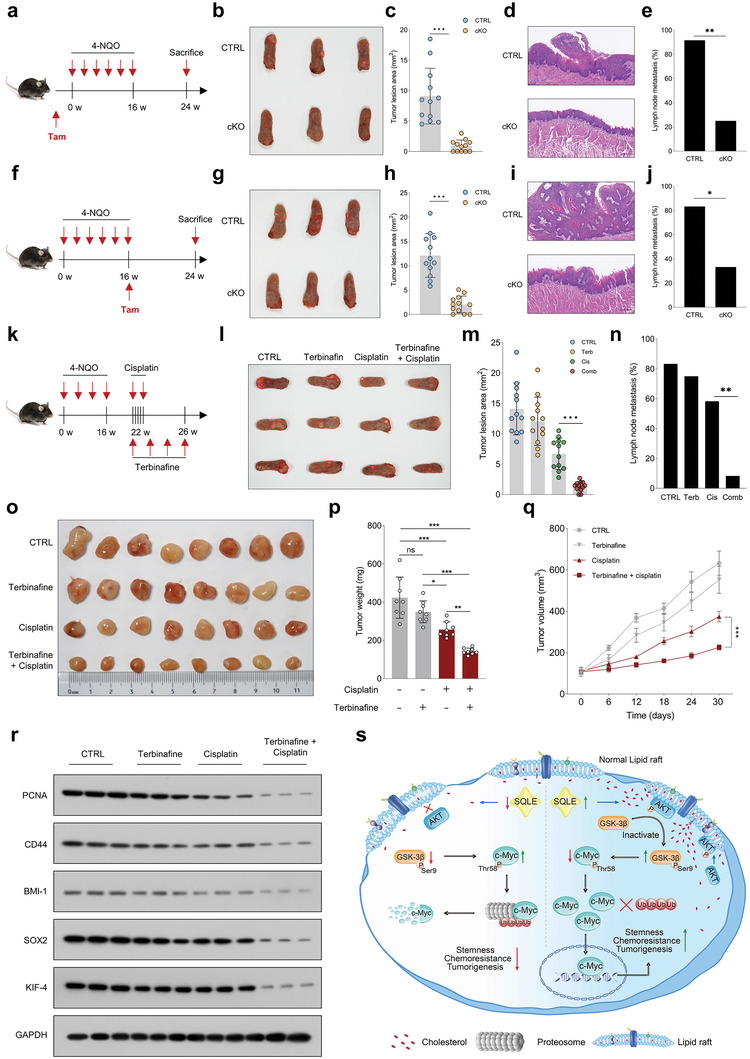
SQLE is crucial for HNSCC initiation and progression, and pharmacological inhibition of SQLE sensitizes cancer cells to cisplatin in vivo. a) Schematic diagram illustrating the determination of *Sqle* conditional KO effects in oral epithelial cells on HNSCC initiation. b,c) Representative images of tongue lesions and tumor lesion area in *Sqle* ^CTRL^ and *Sqle* ^cKO^ mice (*n* = 12). d) Representative histological images of tongue lesions in *Sqle* ^CTRL^ and *Sqle* ^cKO^ mice. e) Metastatic lymph nodes in *Sqle* ^CTRL^ and *Sqle* ^cKO^ groups (*n* = 12). f) Schematic diagram evaluating the effects of *Sqle* conditional KO on HNSCC development. g,h) Representative gross and histological images of tongue lesions in *Sqle* ^CTRL^ and *Sqle* ^cKO^ mice. i,j) Tumor lesion area and metastatic lymph nodes in *Sqle* ^CTRL^ and *Sqle* ^cKO^ groups (*n* = 12). k) Schematic diagram evaluating the combined use of terbinafine and cisplatin in reducing tumor growth in orthotopic mouse model. l) Representative images of tongue lesions from mice with indicated treatments. m,n) Tumor lesion area and metastatic lymph nodes in mice with indicated treatments (*n* = 12). o–q) Effects of terbinafine and cisplatin on suppressing tumor growth in the PDX model (*n* = 8). r) Western blot analyses of PCNA, CD44, SOX2, and KIF4 expression in xenograft tumor tissues subjected to indicated treatments. s) Proposed model depicting the regulation of c‐Myc by SQLE through lipid raft‐localized Akt. Scale bar: 200 µm. Data were presented as mean ± SD. ns (not significant), *p < 0.05, **p < 0.01, ***p < 0.001 for Student's *t*‐test and one‐way ANOVA test.

Given the essential role of SQLE in regulating HNSCC tumorigenesis, we generated a tamoxifen‐mediated conditional *Sqle* gene knockout (KO) mouse model. The schematic diagram illustrating the generation of the cKO model is shown in Figure S18a,b of the Supporting Information, and SQLE expression was scarcely detected in tongue tissues of *Sqle* KO mice following tamoxifen induction (Figure S18c, Supporting Information). Subsequently, we assessed the impact of *Sqle* cKO on HNSCC tumor initiation, with the study design depicted in **Figure** **8**a. Both *Sqle* ^cKO^ and control mice were administered tamoxifen prior to 4‐NQO treatment. After the specified treatments, mice were euthanized, and tongue tissues and lymph nodes were collected for further analysis. We observed a significantly larger tumor lesion area in control mice compared to *Sqle* ^cKO^ mice (Figure 8b,c). Furthermore, histological staining revealed that the majority of control mice developed pronounced tumor lesions, while no evident histological abnormalities were found in *Sqle* ^cKO^ mice (Figure 8d). Additionally, the lymph node metastasis (LNM) rate was markedly higher in *Sqle* ^CTRL^ mice than in *Sqle* ^cKO^ mice (Figure 8e). These data suggest that *Sqle* depletion impedes HNSCC tumor initiation. We then investigated whether *Sqle* cKO affected HNSCC progression, with the study design outlined in Figure 8f. Tamoxifen was administered to the indicated mice following 16 weeks of 4‐NQO treatment, during which tumors formed in the tongue. *Sqle* ^cKO^ mice exhibited a significantly reduced tumor lesion area and more favorable histology compared to *Sqle* ^CTRL^ mice (Figure 8g–i). Moreover, *Sqle* ^cKO^ mice had a notably lower LNM rate than *Sqle* ^CTRL^ mice (Figure 8j). These results demonstrate the critical role of SQLE depletion in halting HNSCC development.

### 2.9 Pharmacological Inhibition of SQLE Enhances Cisplatin Sensitivity in HNSCC Cells In Vivo

Our findings reveal that SQLE is overexpressed in cisplatin‐resistant tumors and its loss restore tumor sensitivity to cisplatin, implicating SQLE as a promising anticancer therapeutic target for cisplatin‐resistant HNSCC. Terbinafine, an FDA‐approved drug that targets SQLE, is widely prescribed for treating fungal infections. Recent evidence has indicated that terbinafine may also exhibit potential anticancer properties.^[^ ^34^ ^]^ Subsequently, we examined the efficacy of pharmacologically targeting SQLE on cisplatin response in an orthotopic mouse model and a PDX mouse model. The study design schematic is illustrated in Figure 8k. In brief, tumor‐bearing mice were randomly assigned to four groups: control vehicle, cisplatin, terbinafine, or cisplatin plus terbinafine. We observed that cisplatin or terbinafine treatment alone partially reduced tumor lesion area and metastatic lymph nodes; however, the combination of cisplatin and terbinafine exhibited the most potent suppressive effects on tumor progression (Figure 8l–n). We then assessed the potential synergistic effect of cisplatin and terbinafine on tumor growth in HNSCC PDX mouse models. No significant changes in tumor growth were observed in terbinafine‐treated mice compared to vehicle‐treated mice. While cisplatin reduced tumor size, weight, and volume in HNSCC PDX mice, the combination of cisplatin and terbinafine resulted in a more robust inhibitory effect than cisplatin alone (Figure 8o–q). Additionally, the combined cisplatin and terbinafine treatment was most effective in reducing the expression of PCNA, SOX2, BMI‐1, SOX2, and KIF4 in the PDX xenografts (Figure 8r). These data provide proof of principle that SQLE‐targeting compounds in combination with cisplatin have a strong synergistic effect on suppressing tumor progression, and targeting SQLE could serve as an effective therapeutic strategy for overcoming cisplatin resistance in HNSCC.

Previous studies suggested that terbinafine might exert its antitumor function independently of SQLE, such as activating AMPK or inhibiting KSR1.^[^ ^29^, ^30^ ^]^ To exclude potential off‐target effects of terbinafine, we performed additional in vivo experiments to examine the impact of terbinafine on the tumorigenic potential of SQLE‐overexpressing SCC‐1^cisR^ cells. The results revealed a significant reduction in tumor size, weight, and volume in the terbinafine‐treated group compared to the control group (Figure S19a–c, Supporting Information). These findings indicate that terbinafine selectively suppresses oncogenic behaviors in SQLE‐overexpressing cells, providing strong evidence that the tumor‐suppressive effects of terbinafine are attributable to SQLE inhibition. Furthermore, we compared the tumorigenic potential of SQLE‐overexpressing SCC‐1^cisR^ cells in both the siSQLE + cisplatin group and the terbinafine + cisplatin group. The results demonstrated that siRNA targeting SQLE produced similar tumor‐reducing effects as terbinafine in SQLE‐overexpressing cells (Figure S19a–c, Supporting Information), supporting the conclusion that terbinafine's antitumor activity primarily targets SQLE. Additionally, our analysis of xenografts revealed a significant reduction in SQLE expression in terbinafine‐treated samples compared to control xenografts (Figure S19d, Supporting Information). Terbinafine also markedly decreased SQLE expression in SCC‐1^cisR^ and SCC‐23^cisR^ cells (Figure S19e, Supporting Information). Collectively, these data support the hypothesis that terbinafine's primary antitumor mechanism is through SQLE inhibition. Our western blot results showed that terbinafine has a minor impact on KSR1 expression (Figure S19f, Supporting Information). Additionally, the level of p‐AMPK was slightly increased following terbinafine treatment in SCC‐1^cisR^ and SCC‐23^cisR^ cells (Figure S19g, Supporting Information). The discrepancies between our results and previous findings could be attributed to differences in cell line models used or variations in the concentration and duration of terbinafine treatment.

## 3 Discussion

Cisplatin‐based chemotherapy remains the cornerstone of therapeutic options for multiple solid tumors, including HNSCC. Nevertheless, a majority of patients with advanced disease inevitably develop acquired resistance through various mechanisms,^[^ ^31^, ^32^ ^]^ underscoring the urgent need for novel strategies to enhance the clinical efficacy of cisplatin‐based chemotherapy. Although impaired cholesterol homeostasis contributes to chemoresistance and the combined use of cholesterol‐lowering drugs appears effective in augmenting cisplatin sensitivity,^[^ ^17^, ^18^ ^]^ the precise role of cholesterol and cholesterol‐related metabolic enzymes in modulating cisplatin resistance in HNSCC has yet to be elucidated. As illustrated in Figure 8s, we have elucidated a novel molecular mechanism by which SQLE contributes to cisplatin resistance and tumorigenesis in HNSCC, using a combination of in vitro and in vivo models, and an in‐house HNSCC cohort. Our findings reveal that the *β*‐catenin/TCF4 complex transcriptionally activates SQLE upon cisplatin treatment, and that SQLE upregulation serves as a prognostic biomarker for survival in HNSCC patients. Furthermore, we demonstrate that SQLE fosters tumorigenic phenotypes and cisplatin resistance by modulating cholesterol levels and maintaining c‐Myc transcriptional activity. The mechanistic understanding of SQLE's role in destabilizing c‐Myc protein through lipid raft cholesterol content modulation and the subsequent impact on Akt and GSK‐3*β* signaling is a significant advancement in the field. Importantly, our study shows that the combined use of terbinafine and cisplatin has a synergistic effect on arresting tumor growth, highlighting the potential therapeutic value of targeting SQLE to enhance cisplatin efficacy in HNSCC treatment.

Abnormal expression of SQLE has been previously associated with tumor progression,^[^ ^33^, ^34^, ^35^ ^]^ and its targeting has proven effective in treating nonalcoholic fatty liver disease‐induced hepatocellular carcinoma.^[^ ^26^ ^]^ Recent research also demonstrated SQLE's role in promoting colorectal cancer tumorigenesis through regulation of the gut microbiota‐metabolite axis.^[^ ^36^ ^]^ However, the involvement of SQLE in therapy resistance in cancer and the underlying molecular mechanisms have not been fully understood. Our study provides novel insights by revealing the critical role of aberrantly upregulated cholesterol content in modulating cisplatin resistance in HNSCC cells, identifying SQLE as the most significantly increased cholesterol‐related metabolic enzyme in cisplatin‐resistant xenograft tissues. Furthermore, we uncovered the previously unknown mechanism that the *β*‐catenin/TCF4 complex transcriptionally activates SQLE, which drives drug resistance. Importantly, we established that SQLE overexpression serves as a predictor of unfavorable prognosis and chemoresistance responses in the HNSCC cohort. As a result, incorporating SQLE alongside other known markers, such as EGFR and CD44, in conjunction with clinical parameters, has the potential to enhance the clinical outcome of HNSCC patients.

Our experimental results offer compelling in vitro and in vivo evidence that SQLE depletion significantly inhibits oncogenic phenotypes and enhances cisplatin sensitivity in cisplatin‐resistant HNSCC cells. These findings suggest that SQLE could be a critical therapeutic target for overcoming acquired cisplatin resistance. We observed that SQLE depletion minimally impacted the expression of DNA damage response‐related proteins, implying that SQLE inhibition might not sensitize cells to cisplatin through impaired DNA damage repair. This outcome may be attributed to the use of a cisplatin‐resistant cell line model, which has been reported to possess significantly higher DNA damage repair capacity compared to parental counterparts.^[^ ^37^ ^]^ CSCs, a small subpopulation of tumor‐initiating cells with self‐renewal potential and high adaptive abilities, play a crucial role in drug resistance, tumor growth, recurrence, and metastasis.^[^ ^38^, ^39^ ^]^ Although chemotherapy drugs like cisplatin can effectively eliminate rapidly proliferating cancer cells and reduce tumor mass, CSC persistence often leads to tumor relapse and treatment failure.^[^ ^40^ ^]^ Our study discovered that SQLE governs cancer stemness in primary HNSCC cells, as demonstrated by in vitro and in vivo limiting dilution assays. Since SQLE is essential for maintaining cholesterol homeostasis and abnormal lipid metabolism is a hallmark of CSCs, we hypothesized that SQLE might promote malignant phenotypes and cancer stemness by modulating cholesterol levels. Intriguingly, cholesterol repletion partially restored the tumorigenic phenotypes suppressed by SQLE depletion, indicating that SQLE regulates HNSCC tumorigenesis by maintaining cholesterol balance in cancer cells.

In an effort to elucidate the molecular mechanism underlying SQLE's tumor‐promoting role in HNSCC, our study employed unbiased transcriptomic analyses, which revealed a significant suppression of c‐Myc‐targeted genes in cancer cells following SQLE depletion. As c‐Myc, a transcription factor frequently overexpressed in human cancers, is known for its role in maintaining self‐renewal and drug‐resistant properties in CSCs,^[^ ^41^, ^42^ ^]^ these findings shed new light on the relationship between SQLE and c‐Myc. Our study is the first to demonstrate that SQLE regulates c‐Myc protein levels by mediating its polyubiquitylation and proteolysis, and has little effect on the expression of c‐Myc mRNA. Additionally, we explored the role of lipid rafts, small dynamic membrane microdomains enriched in cholesterol and regulatory molecules,^[^ ^43^ ^]^ in this context. Since SQLE is crucial for cholesterol homeostasis in HNSCC cells, we hypothesized that it might influence cholesterol content in lipid rafts, thereby regulating the lipid raft‐associated signaling cascade. Our study provides novel evidence that SQLE inhibition reduces cholesterol levels in lipid rafts, inhibits Akt binding, and affects c‐Myc stability through increased GSK‐3*β* activity. Notably, cholesterol repletion reinstated the expression of lipid raft‐localized Akt in HNSCC cells experiencing SQLE depletion. Subsequently, we ascertained that c‐Myc functioned as a downstream target of SQLE, further elucidating the molecular relationship. Given the challenges in directly targeting the “undruggable” c‐Myc protein structure,^[^ ^44^ ^]^ our study highlights the potential of targeting SQLE as a novel, practical, and innovative approach for inhibiting c‐Myc activity in cancer treatment, thus emphasizing the significance of our findings in the field of cancer therapeutics.

Notably, our study demonstrates that *Sqle* knockout not only precludes HNSCC initiation but also effectively impedes its progression. These results offer valuable insights into the crucial role of SQLE and cholesterol homeostasis in HNSCC tumorigenesis. Furthermore, we evaluated the therapeutic potential of combining terbinafine with cisplatin in impeding tumor growth across various preclinical models. Our findings indicate that the concomitant administration of terbinafine and cisplatin exhibits robust synergistic therapeutic efficacy in attenuating tumor growth. Consequently, targeting SQLE using small chemical compounds could represent a more efficacious and pragmatic approach for surmounting cisplatin resistance in HNSCC.

Several limitations of our study should be acknowledged. First, our investigation did not assess the impact of SQLE on the tumor microenvironment, immune response, or interactions with other stromal cells. A deeper understanding of SQLE's role in these aspects could provide valuable insights into tumor biology and inform potential therapeutic strategies. Second, our study did not explore the possible role of SQLE in other types of cancers. The mechanism of SQLE‐mediated c‐Myc regulation through cholesterol‐dependent pathways might be relevant to other malignancies, and further research in this area could help broaden the applicability of our findings. Lastly, there is an urgent need to develop novel small molecules that target SQLE with higher specificity than terbinafine to expedite the translation of our findings into clinical practice.

In summary, our findings unveil a previously undiscovered and critical mechanism underlying SQLE‐mediated c‐Myc activation in HNSCC. The essential role of SQLE in maintaining cholesterol homeostasis, cancer cell stemness, HNSCC initiation and progression, and cisplatin resistance renders it a promising molecular target for HNSCC treatment. Ultimately, we offer valuable insights into the ways metabolic vulnerability influences chemoresistance and provide compelling preclinical evidence supporting the targeting of SQLE to enhance chemotherapy response.

## 4 Experimental Section

### Materials

The following antibodies were purchased from Proteintech Group Inc (Chicago, IL, USA): anti‐SQLE (#12544‐1‐AP, 67206‐1‐Ig) (for western blotting, 1:2000 dilution), anti‐GAPDH (#10494‐1‐AP) (for western blotting, 1:6000 dilution), anti‐CD44 (#15675‐1‐AP) (for western blotting, 1:4000 dilution and IHC, 1:100 dilution), anti‐BMI‐1 (#10832‐1‐AP) (for western blotting, 1:1000 dilution), anti‐c‐Myc (#10828‐1‐AP) (for western blotting, 1:2000 dilution), anti‐PCNA (#10205‐2‐AP) (for western blotting, 1:5000 dilution), anti‐PTEN (#22034‐1‐AP) (for western blotting, 1:2000 dilution), anti‐TCF4 (#22337‐1‐AP) (for western blotting, 1:3000 dilution), anti‐*β*‐catenin (#51067‐2‐AP) (for western blotting, 1:5000 dilution), anti‐Flag (#20543‐1‐AP) (for western blotting, 1:4000 dilution and IP, 1:100 dilution), anti‐*β*‐actin (#20536‐1‐AP) (for western blotting, 1:6000 dilution), anti‐AKT (#10176‐2‐AP) (for western blotting, 1:2000 dilution and IP, 1:50 dilution), anti‐GSK‐3*β* (#22104‐1‐AP) (for western blotting, 1:3000 dilution and IP, 1:50 dilution), and anti‐GNAI2 (#11136‐1‐AP) (for western blotting, 1:1000 dilution). The following antibodies were purchased from Abcam (Cambridge, UK): anti‐V5 (#ab27671) (for western blotting, 1:2000 dilution), anti‐SOX2 (#ab924 94) (for western blotting, 1:2000 dilution). The primary antibodies against p‐GSK‐3*β* S9 (#9336) (for western blotting, 1:1000 dilution), p‐AKT S473 (#9271) (for western blotting, 1:1000 dilution). A rabbit antibody against Flot2 (#PA5‐79268) (for western blotting, 1:1000 dilution) was purchased from Thermo Fisher Scientific, and anti‐KIF‐4 was from GeneTex, Inc (Irvine, CA, USA). Cisplatin (#HY‐15925), water‐soluble cholesterol (#HY‐N0322A), terbinafine (#HY‐17395A), and NB598 (HY‐16343) were purchased from MedChem Express (Monmouth Junction, NJ, USA). 4‐NQO (#N8141) and tamoxifen (#T5648) were from Sigma‐Aldrich (Burlington, MA, USA).

### Cell Culture

The UMSCC‐1 (SCC‐1) and UTSCC‐23 (SCC‐23) cell lines were obtained from the University of Michigan, while CAL‐27 (Cat#: CRL‐2095) was procured from the American Type Culture Collection. The HN30 cell line was kindly provided by Dr. Ran at the First Affiliated Hospital of Sun Yat‐sen University. All cell lines underwent authentication through short tandem repeat DNA fingerprinting and tested negative for mycoplasma contamination. Cisplatin‐resistant cell lines, SCC‐1^cisR^, SCC‐23^cisR^, and CAL‐27^cisR^, were generated as previously described.^[^ ^45^ ^]^ All cell lines were cultured in high‐glucose DMEM medium supplemented with 10% FBS and 100 units mL^−1^ penicillin/streptomycin and maintained at 37 °C in a humidified incubator with 5% CO_2_. Cells were passaged for fewer than six months after resuscitation. hDPSCs and hPDLSCs were isolated and cultured as previously described by the group.^[^ ^46^, ^47^ ^]^

### Patients and Clinical Samples

The research protocol for reusing clinical specimens and medical information was approved by the Ethics Committee of the First Affiliated Hospital of Sun Yat‐sen University (IRB Number: 2022–056), and all procedures adhered to the Declaration of Helsinki guidelines. Written informed consent was obtained from all subjects. Inclusion criteria for the internal HNSCC cohort included histopathological and clinical diagnoses of HNSCC and no prior chemotherapy or radiation therapy. Exclusion criteria involved patients with multiple primary malignancies or missing clinical data. All tissue specimens were snap‐frozen in liquid nitrogen and stored at −80 °C without thawing until needed for protein extraction or underwent standard formalin‐fixed and paraffin‐embedded procedures. For HNSCC patients receiving induction chemotherapy, the regimen included docetaxel 75 mg m^−2^ on day 1, cisplatin 75 mg m^−2^ on day 1, and 5‐fluorouracil 750 mg m^−2^ via infusion for 120 h on days 1–5, with cycles administered every three weeks for a total of 2 cycles. Patients underwent regular follow‐up after surgery, and tumor responses to neoadjuvant chemotherapy were assessed by clinical evaluation and imaging according to the Response Evaluation Criteria in Solid Tumors guidelines. CR was defined as the disappearance of all target lesions without evidence of tumors elsewhere. PR was defined as a minimum 30% decrease in the sum of the longest diameters of target lesions. PD was defined as at least a 20% increase in the sum of the longest diameters of target lesions, referencing the smallest recorded sum since treatment began or the appearance of new lesions. SD was defined as neither sufficient shrinkage for PR nor sufficient increase for PD, using the smallest recorded sum of the longest diameters since treatment initiation. Primary cancer cells from HNSCC samples were isolated following the previously published procedures.^[^ ^45^ ^]^ The detailed information of the in‐house HNSCC cohort was provided in Table S1 of the Supporting Information.

### Plasmids, SiRNAs, Cell Transfection, and Lentivirus Production

SiRNAs targeting SQLE, TCF4, and *β*‐catenin were synthesized by RiboBio (Guangzhou, China), and transient siRNA transfections were performed using Lipofectamine RNAiMAX (Thermo Fisher Scientific). TCF4 and *β*‐catenin overexpression plasmids were obtained from GenePharma (Shanghai, China), and transient plasmid DNA transfections were carried out with Lipofectamine 3000 (Thermo Fisher Scientific) following the manufacturer's guidelines. Short hairpin RNAs (shRNAs) targeting SQLE were integrated into the LV3‐pGLV‐h1‐GFP‐puro plasmid (GenePharma) to construct recombinant lentiviral vectors. Full‐length human SQLE cDNA was cloned into the pGCL‐GFP plasmid (Genechem, Shanghai, China) to produce the SQLE overexpression construct. Recombinant plasmids containing transgenes and packaging plasmids were cotransfected into HEK‐293T cells to generate lentiviruses. Viruses were harvested 72 h post‐transfection, and cells were subsequently infected with concentrated viruses. Stable cell lines were established after antibiotic selection. c‐Myc shRNA lentiviral particles were sourced from Santa Cruz Biotechnology (Santa Cruz, CA, USA). Oligo sequences utilized in this study can be found in Table S2 of the Supporting Information.

### RNA Extraction and qRT‐PCR Analysis

Total RNA was extracted from cells using the Quick‐RNA kit (Zymo Research Corp, #R1054). Two micrograms of RNA were reverse transcribed into cDNA with SuperScript III First‐Strand Synthesis SuperMix (Thermo Fisher Scientific, #18080‐400), and cDNA was then employed as a template for qRT‐PCR using Light Cycler 480@ SYBR Green I MasterMix (Roche, #04707516001) on a CFX96 Real‐Time PCR detection system (Bio‐Rad). The primers for qRT‐PCR are listed in Table S2 of the Supporting Information.

### Western Blotting

Proteins were extracted from cell culture or tissues using RIPA lysis buffer (Beyotime, #P0013E) containing protease and phosphatase inhibitor cocktail (Thermo Fisher Scientific, #78440). Proteins were separated on 4–20% gradient gels via electrophoresis and transferred to PVDF membranes (Bio‐Rad, #1620177) with the Trans‐Blot Turbo transfer system (Bio‐Rad). After blocking with protein‐free rapid blocking buffer (Epizyme, #PS108P) for 10 min at room temperature, membranes were incubated with primary antibodies overnight at 4 °C, followed by HRP‐linked secondary antibodies for 1 h at room temperature. Amersham ECL Prime Western Blotting Detection Reagent (Cytiva, #RPN2236) was used to visualize protein bands, and band intensity was quantified using ImageJ (National Institutes of Health).

### Cell Proliferation Assay

Cells subjected to indicated treatments were seeded in 96‐well plates at a density of 3000 cells per well, and cell viability was measured for four consecutive days using the MTT assay. For the colony formation assay, cells were seeded in 6‐well plates at a density of 1000 cells per well and incubated for two weeks. Colonies were then fixed, stained with 0.1% crystal violet, and counted.

### Sphere Formation Assay

The sphere formation assay was conducted according to the previously published methods. Briefly, cells were seeded in ultralow attachment plates and maintained in medium supplemented with 1% B27 (Invitrogen), 1% N2 (Invitrogen), and growth factors at 37 °C.

### ALDEFLUOR Assay

The effects of SQLE depletion on ALDH activity in HNSCC cells were assessed using the ALDEFLUOR Kit (STEMCELL Technologies, #01700) following the manufacturer's instructions. For a negative control, an aliquot of cells was treated with 50 mm ALDH inhibitor diethylaminobenzaldehyde. Samples were kept on ice and shielded from light until analysis with the DxFLEX flow cytometer (Beckman Coulter).

### Annexin V/PI Apoptosis Assay

For apoptosis detection, cells subjected to the indicated treatments were trypsinized, harvested, and placed into FACS tubes. After washing with ice‐cold PBS, cell pellets were resuspended in 100 µL of binding buffer (10 mм HEPES, pH 7.4, 140 mм NaCl, 2.5 mм CaCl_2_) and incubated with 5 µL of annexin V‐APC (Thermo Fisher Scientific, #88800772) for 15 min at 25 °C in the dark. Samples were then washed and resuspended in binding buffer before adding 5 µL of propidium iodide to each tube. All samples were analyzed within 1 h using the DxFLEX flow cytometer (Beckman Coulter, Inc.).

### Cholesterol Measurement

Cells or tissues subjected to the indicated treatments were collected, and cholesterol concentrations were measured using the Cholesterol/Cholesteryl Ester Quantitation Assay kit (ab65359, Abcam) following the manufacturer's instructions.

### Sucrose Density Gradient Fractionation

Lipid rafts were extracted from cells treated as indicated using the sucrose density gradient centrifugation technique. Briefly, cell lysates were overlaid sequentially on lysis buffer containing different sucrose concentrations to form a discontinuous sucrose gradient. The samples were centrifuged at 44 000 rpm at 4 °C for 18 h. Twelve fractions were collected from the top to the bottom of the centrifuged liquid and subsequently subjected to western blotting.

### CHX Chase Assay

Transfected cells were treated with CHX (50 mg mL^−1^) for the indicated time periods, and cell lysates were collected for western blot analysis.

### Immunoprecipitation

Cells subjected to the indicated treatments were lysed in RIPA buffer (Beyotime) at 4 °C, and the cell lysates were incubated with corresponding antibodies for 12 h at 4 °C to form antibody–protein complexes. The complexes were immunoprecipitated with protein A/G magnetic beads at 4 °C overnight to form bead–antibody complexes. After extensive washing with elution buffer, the protein complexes were boiled and analyzed by western blotting.

### IHC Assay

Following deparaffinization, rehydration, endogenous peroxidase deactivation, antigen retrieval, and protein blocking, tissue sections were incubated with various primary antibodies overnight at 4 °C, followed by incubation with HRP‐conjugated secondary antibodies. The DAB substrate solution was used to visualize the results. The IHC results were quantitatively scored under a microscope by multiplying the staining intensity and the percentage of stained cells. An H score was assigned to each slide according to the following formula: 3 × percentage of strongly stained + 2 × percentage of moderately stained + 1 × percentage of weakly stained + 0 × percentage of unstained. The assessment was independently performed by two pathologists who were blinded to the clinical information.

### ChIP‐qPCR

The ChIP assay was performed using the EZ ChIP Chromatin Immunoprecipitation Kit (Millipore, Billerica, MA, USA), following a previously described protocol.^[^ ^45^ ^]^ Briefly, DNA–protein cross‐links were generated by fixing the cell samples with 1% formaldehyde for 10 min at room temperature. After washing three times with PBS, the samples were lysed in RIPA buffer and then subjected to sonication to fragment the genomic DNA. The sheared chromatin was immunoprecipitated with an anti‐TCF4 antibody overnight at 4 °C. PCR was subsequently performed to identify the associated DNA sequences.

### Luciferase Reporter Assay

The wild‐type (pGL3‐SQLE‐wt) or mutant ACTN1 (pGL3‐SQLE‐mut) luciferase reporter plasmids were transfected into cells with the specified modifications using Lipofectamine 3000 transfection reagent (Invitrogen), following the manufacturer's instructions. Relative luciferase activity was measured 24 h post‐transfection using the Dual‐Luciferase Reporter Assay System (Promega, Madison, WI, USA).

### RNA Sequencing

Sequencing was performed on the NovaSeq 6000 platform at Majorbio Bio‐Pharm Technology Co., Ltd. (Shanghai, China). The RNA‐seq transcriptome library was prepared using the Illumina TruSeq RNA Sample Preparation Kit (Illumina, San Diego, USA) according to the manufacturer's recommendations. Raw paired‐end reads were trimmed and quality‐checked using SeqPrep (https://github.com/jstjohn/SeqPrep) and Sickle (https://github.com/najoshi/sickle) with default parameters. The HISAT pipeline (http://www.ccb.jhu.edu/software/hisat/) was employed to align reads against the reference genome. Mapped reads for each sample were assembled using StringTie (http://ccb.jhu.edu/software/stringtie/). Differentially expressed genes (DEGs) were identified using DEGseq, with a cutoff of absolute FC ≥ 2 and an adjusted *p*‐value ≤ 0.001.

### Bioinformatic Analysis

Public repositories containing genome‐wide gene expression profiling comparisons between tumor tissues and ANTs/normal tissues/precancerous lesions were retrieved from the Gene Expression Omnibus database (https://www.ncbi.nlm.nih.gov/geo/). The accession numbers were GSE127165, GSE37991, GSE30784, GSE31056, GSE85446, GSE2837, GSE41613, and GSE26549. The RNA‐seq transcriptome data and corresponding clinical information for the TCGA HNSCC cohort were obtained from The National Cancer Institute Genomic Data Commons (https://gdc.cancer.gov/). The X‐tile software (https://medicine.yale.edu/lab/rimm/research/‐software/) was utilized to identify the most optimal cutoff point for dividing HNSCC patients into high and low SQLE expression groups. For GSEA, HNSCC patients were divided into *SQLE*‐high and *SQLE*‐low expression groups based on median *SQLE* expression. GSEA software was then used to determine if predefined gene sets were statistically significant.

### Generation of Cisplatin‐Resistant HNSCC Tumors

All the animal experiments were approved by the Institutional Animal Care and Use Committee at Southern Medical University (SMUL2022003). To generate cisplatin‐resistant xenografts, 6‐week‐old male BALB/c nu/nu mice were subcutaneously inoculated with 2 × 10^6^ SCC‐1 cells. Mice were randomly assigned to two groups: vehicle and cisplatin (cisplatin 5 mg kg^−1^ intraperitoneally per week). After 2–3 weeks postinjection, indicated treatments were initiated when tumors became palpable. For the cisplatin‐treated group, mice responding to treatment were sacrificed, and tumor samples were collected and designated as the cisplatin‐sensitive group. The remaining mice continued to receive cisplatin treatment until tumors began to regrow, and these tumor samples were designated as the cisplatin‐resistant group.

### Subcutaneous Tumor Model

To assess the effects of SQLE depletion on tumor growth and cisplatin resistance in HNSCC, 2 × 10^6^ SCC‐1^cisR^ or SCC‐23^cisR^ cells with the indicated modifications were subcutaneously injected into 6‐week‐old male BALB/c nu/nu mice. Groups were assigned as follows: shCTRL, shSQLE #1, shSQLE #2, shCTRL + cisplatin, shSQLE #1 + cisplatin, and shSQLE #2 + cisplatin. Four weeks after cancer cell injection, all mice were sacrificed, and tumor samples were collected for further analysis.

### In Vivo Limiting Dilution Tumor Initiation Assay

A limiting dilution transplantation assay was performed to determine the effects of SQLE depletion or overexpression on HNSCC tumorigenesis. EpCAM^+^/ALDH^+^ primary HNSCC cells were isolated from HNSCC tissues following the previously published procedures.^[^ ^45^ ^]^ Cells were infected with the indicated lentiviruses, and mice were subsequently transplanted with decreasing numbers of primary HNSCC cells with indicated modifications. Changes in the tumor‐initiating capacity of primary HNSCC cells following SQLE depletion or overexpression were analyzed using the ELDA software (http://bioinf.wehi.edu.au/software/elda/).

### PDX Mouse Model

The PDX model was utilized to evaluate the synergistic therapeutic efficacy of terbinafine and cisplatin, with the PDX xenografts being nonresistant to cisplatin. Primary surgical tumor samples were cut into 1–3 mm^3^ pieces and subcutaneously transplanted into the flanks of NOD/SCID/IL2r*γ*null (NSG) mice. Tumor growth and animal health status were monitored by caliper measurements every three days. Mice were euthanized and sacrificed when the tumor size exceeded 1000 mm^3^, and tumors were harvested and re‐implanted into the next generation (P0, P1, P2). To evaluate the potential synergistic effects of terbinafine and cisplatin, PDX‐bearing mice were given the indicated treatment when tumor size reached 100 mm^3^. Groups were assigned as follows: CTRL, terbinafine, cisplatin, and terbinafine + cisplatin.

### Orthotopic HNSCC Mouse Model

An orthotopic HNSCC mouse model was constructed to assess the combined therapeutic effects of terbinafine and cisplatin. Briefly, six‐week‐old C57BL/6 mice were treated with 4‐NQO in drinking water (50 µg mL^−1^) for 16 weeks and then received regular drinking water for another 10 weeks. Mice were randomly assigned to four groups: CTRL, terbinafine, cisplatin, and terbinafine + cisplatin. Mice received the indicated treatments at week 22 and were sacrificed at week 26. Tumor lesion area was found in the tongue and lymph node. The cervical lymph nodes were dissected and fixed in paraformaldehyde. The specimens were then embedded in paraffin and sectioned. Subsequently, the sections of cervical lymph nodes were subjected to immunostaining using anti‐pan cytokeratin antibody. Finally, the percentage of lymph nodes with metastasis was calculated based on the immunostaining results.

### Conditional Knockout Mouse Model

*K14‐CreER^T2^* mice were purchased from The Jackson Laboratory (Bar Harbor, ME, USA). *Sqle* ^fl/fl^ mice were obtained from Cyagen Biosciences (Guangzhou, China). Male and female *K14‐CreER^T2^*; *Sqle^wt/fl^* mice were used for breeding *K14‐CreER^T2^*; *Sqle^wt/wt^* (*Sqle* ^CTRL^) and *K14‐CreER^T2^; Sqle^fl/fl^* (*Sqle* ^cKO^) mice. To determine the effects of *Sqle* depletion on HNSCC tumor initiation, tamoxifen was administered to the transgenic mice at a dose of 1 mg day^−1^ for five consecutive days before 4NQO treatment. Similarly, tamoxifen was administered at a dose of 100 mg kg^−1^ of body weight for five consecutive days, starting at 16 weeks after 4NQO treatment, to assess the impact of *Sqle* depletion on HNSCC tumor progression.

### Statistical Analysis

Data analyses in this study were conducted using GraphPad Prism 9.0 (GraphPad Software, San Diego, CA, USA). Results are presented as the mean ± standard deviation (SD) based on experiments conducted at least in triplicate. Sample sizes (*n*) vary from 3 to 102, contingent on the particular condition being assessed. Differences between two groups were analyzed using Student's *t*‐test, while one‐way ANOVA was employed to assess differences among multiple groups. Survival curves were plotted using the Kaplan–Meier method and compared via the log‐rank test. Figure legends detail the specific statistical approaches and data presentation. A *p*‐value < 0.05 was considered statistically significant (*p < 0.05; **p < 0.01; ***p < 0.001; ****p < 0.0001)

## Conflict of Interest

The authors declare no conflict of interest.

## Supporting information

Supporting Information

## Data Availability

The data that support the findings of this study are available from the corresponding author upon reasonable request.
