# Supplementary material for: Targeting Squalene Epoxidase Confers Metabolic Vulnerability and Overcomes Chemoresistance in HNSCC
Source: Adv Sci (Weinh). 2023 Jul 25;10(27):2206878. doi: 10.1002/advs.202206878 (PMC10520660; doi:10.1002/advs.202206878)
Supplement: Supplementary file 1 — Supporting Information [file ADVS-10-2206878-s001.pdf]

ADVANCED SCIENCE
Open Access

Supporting Information

for *Adv. Sci.*, DOI 10.1002/advs.202206878

Targeting Squalene Epoxidase Confers Metabolic Vulnerability and Overcomes Chemoresistance in HNSCC

Xinyuan Zhao, Bing Guo, Wenjuan Sun, Jinhua Yu and Li Cui**

ADVANCED SCIENCE
Open Access

Supporting Information

for *Adv. Sci.*, DOI 10.1002/advs.202206878

Targeting Squalene Epoxidase Confers Metabolic Vulnerability and Overcomes Chemoresistance in HNSCC

Xinyuan Zhao, Bing Guo, Wenjuan Sun, Jinhua Yu and Li Cui**

Supporting Information

Targeting squalene epoxidase confers metabolic vulnerability and overcomes chemoresistance in HNSCC

Xinyuan Zhao[a], Bing Guo[b], Wenjuan Sun[c], Jinhua Yu[d], Li Cui[a,e]**

[a]Stomatological Hospital, School of Stomatology, Southern Medical University, Guangzhou, 510280, China

[b]Department of Dentistry, the First Affiliated Hospital, Sun Yat-Sen University, Guangzhou, 510080, China

[c]Department of Stomatology, The Third Affiliated Hospital, Sun Yat-sen University, Guangzhou, 510630, China.

[d]Department of Endodontics, Jiangsu Key Laboratory of Oral Diseases, Affiliated Hospital of Stomatology, Nanjing Medical University, Nanjing, 210029, China

[e]Division of Oral Biology and Medicine, School of Dentistry, University of California, Los Angeles, Los Angeles, 90095, California, United States

*Jinhua Yu Email: yujinhua@njmu.edu.cn

*Li Cui Email: licui@smu.edu.cn

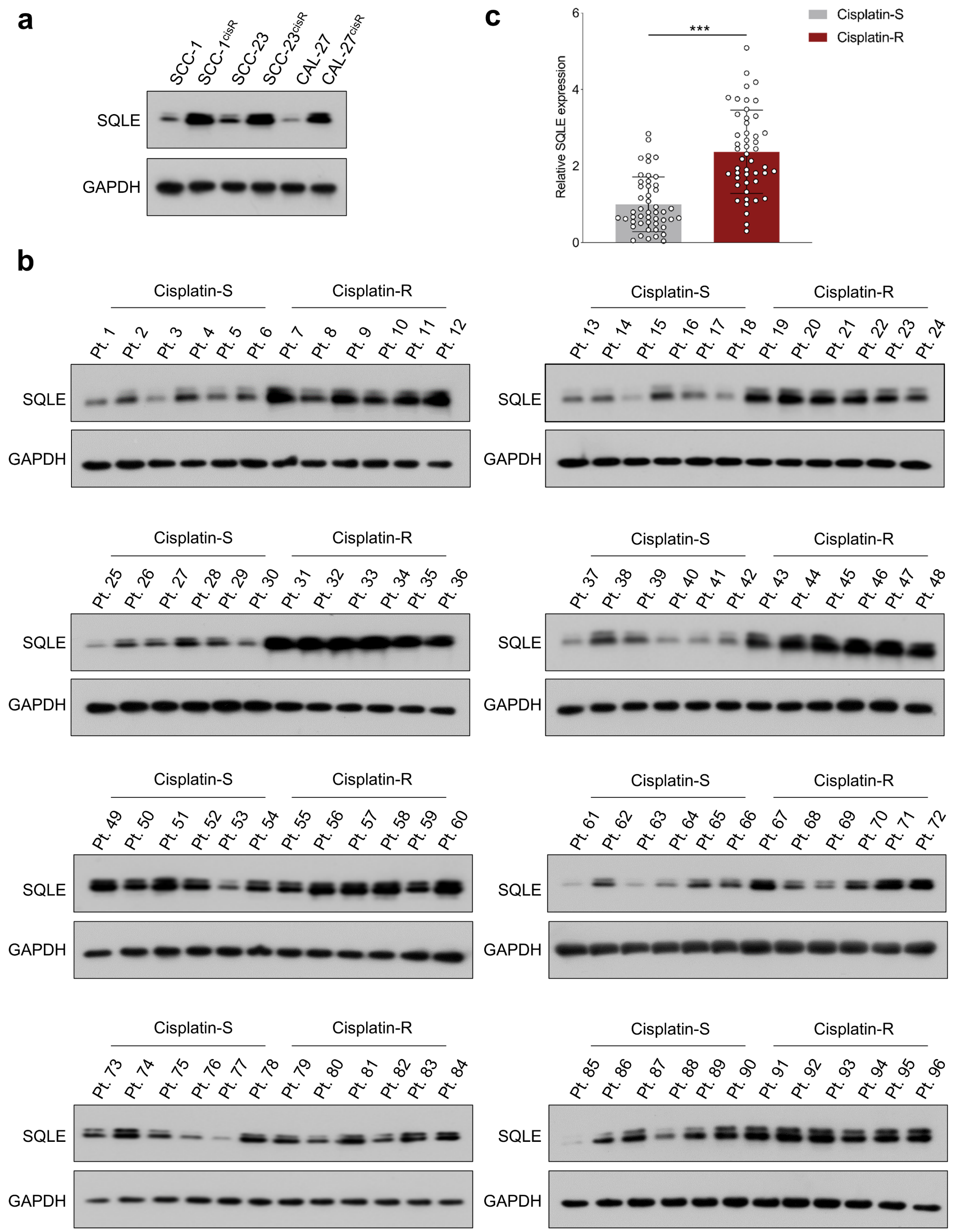

Figure S1. Overexpression of SQLE in cisplatin-resistant cell lines and tumor tissues. a) Western blot analysis comparing SQLE expression levels in cisplatin-resistant HNSCC cell lines to their parental counterparts. b,c) Western blot analysis of SQLE expression in cisplatin-sensitive (n=48) and cisplatin-resistant (n=48) tumor tissues. Data was presented as mean ± SD. ***$p < 0.001$ for Student's t-test.

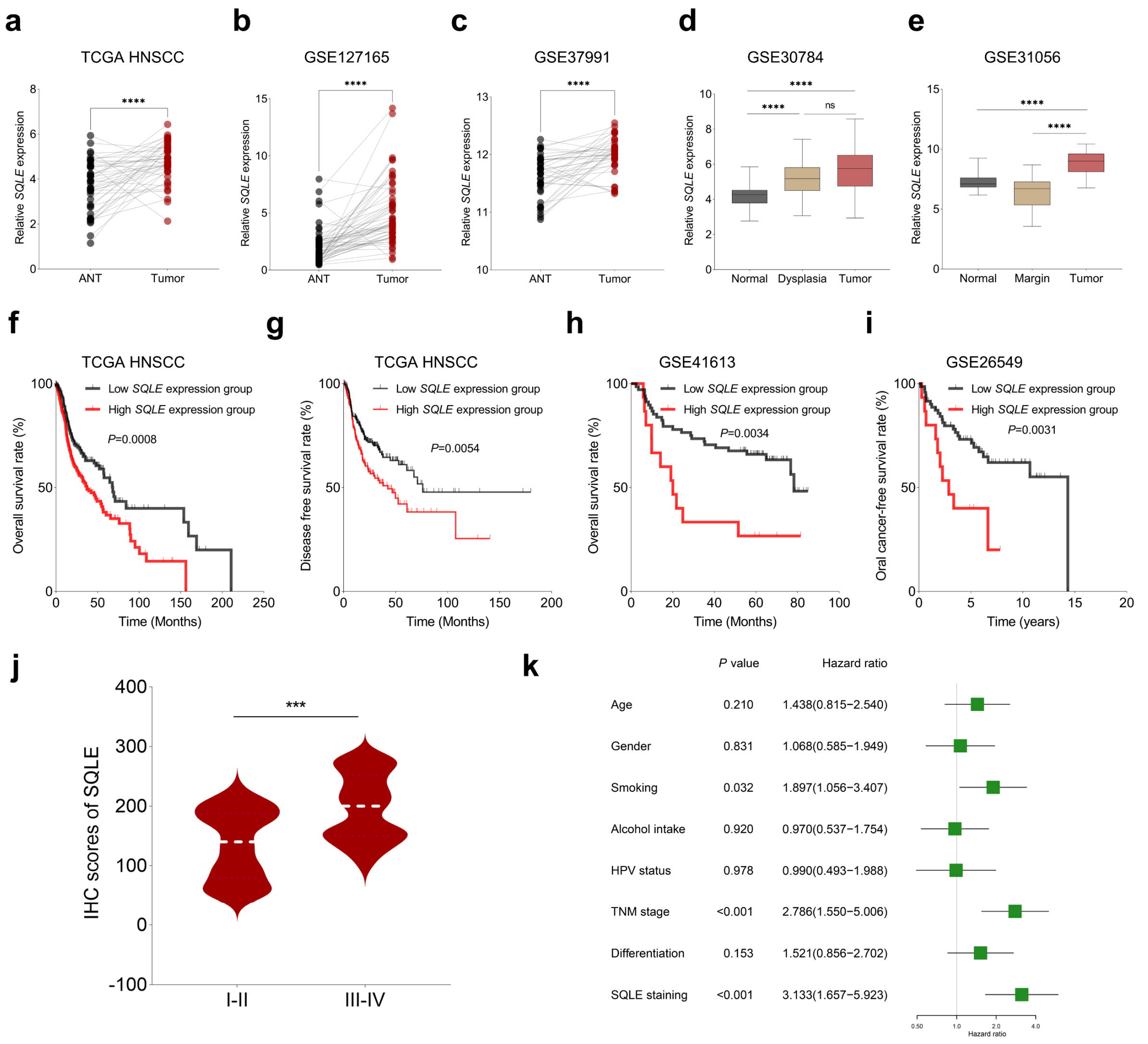

Figure S2. Association of SQLE overexpression with unfavorable clinical outcomes in multiple independent HNSCC cohorts. a-c) Comparison of *SQLE* expression levels in tumor tissues and ANTs across TCGA HNSCC, GSE127165, and GSE37991 datasets. d) *SQLE* expression patterns in normal tissues, dysplasia tissues, and tumor tissues within the GSE30784 dataset. e) *SQLE* expression levels in normal tissues, margin tissues, and tumor tissues in the GSE31056 dataset. f-h) Kaplan-Meier analyses of overall survival or disease-free survival for HNSCC patients stratified by *SQLE* expression levels in TCGA HNSCC and GSE41613 datasets; group differences assessed using the log-rank test. i) Kaplan-Meier analysis of oral cancer-free survival time in patients with oral preneoplastic lesions, grouped based on *SQLE* expression in the GSE26549 dataset; group differences assessed using the log-rank test. j) IHC scores of SQLE in advanced-stage tumor tissues compared to early-stage tumor tissues in our in-house cohort (n=102). k) Univariate analysis of risk factors for predicting overall survival in HNSCC patients (n=102). Data was presented as mean ± SD. ns (not significant), ****p* < 0.001, *****p* < 0.0001 for Student's t-test and one-way ANOVA test.

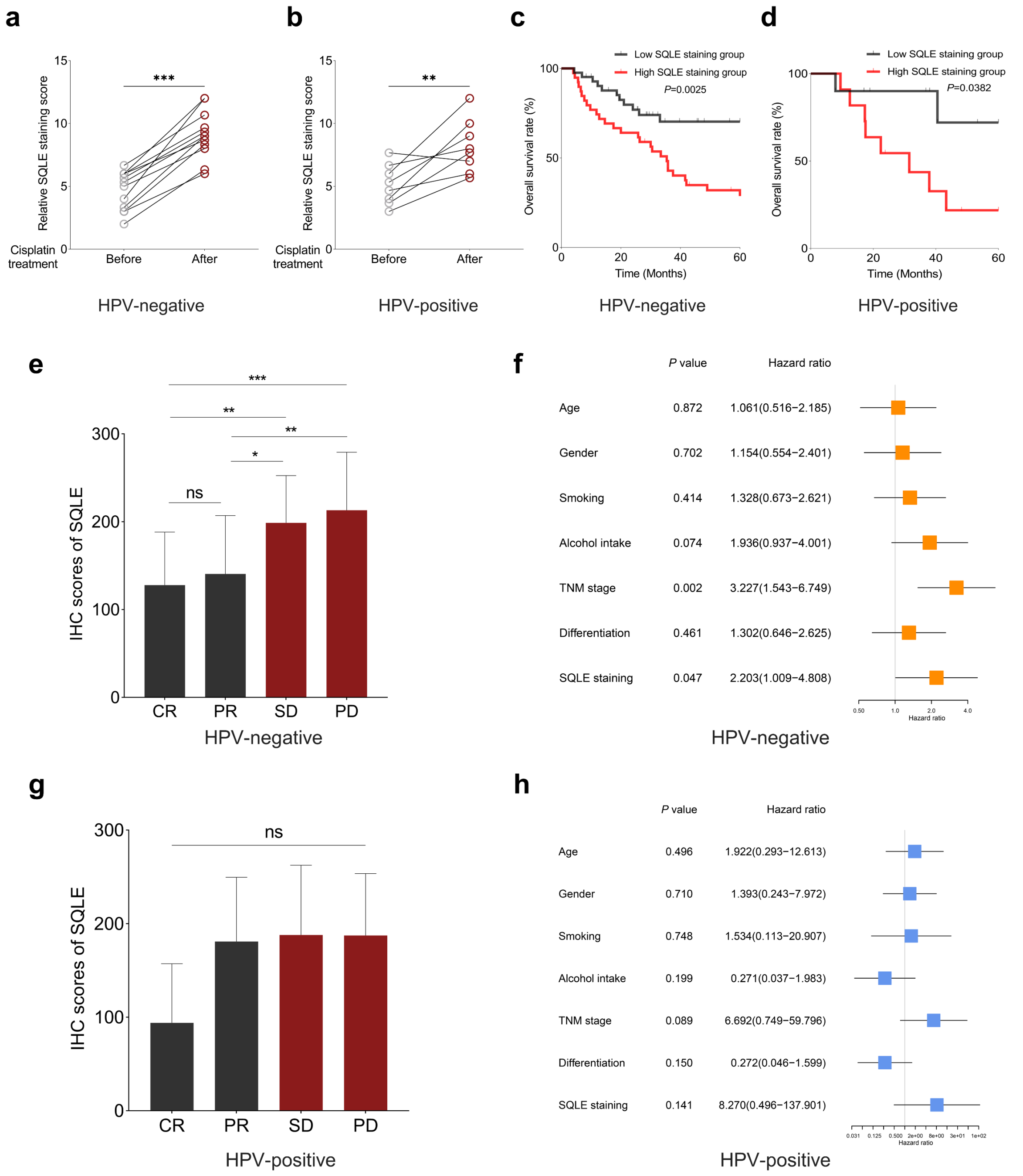

Figure S3. Clinical implications of SQLE expression in HPV-positive and HPV-negative HNSCC subtypes, and its relationship with treatment responses and survival outcomes. a,b) SQLE staining intensity in HPV-positive (n=8) and HPV-negative (n=12) HNSCC tissues before and after cisplatin treatment. c,d) Association between SQLE staining intensity and overall survival in HPV-positive (n=21) and HPV-negative HNSCC (n=81) subgroups. e) Relationship between SQLE intensity and therapeutic responses in the HPV-negative HNSCC cohort (n=81). f) Multivariate analysis identifying independent risk factors for the HPV-negative HNSCC cohort (n=81). g) Relationship between SQLE intensity and therapeutic responses in the HPV-positive HNSCC cohort (n=21). h) Multivariate analysis identifying independent risk factors for the HPV-positive HNSCC cohort (n=21). Data was presented as mean ± SD. ns (not significant), *$p < 0.05$, **$p < 0.01$, ***$p < 0.001$ for Student's t-test and one-way ANOVA test.

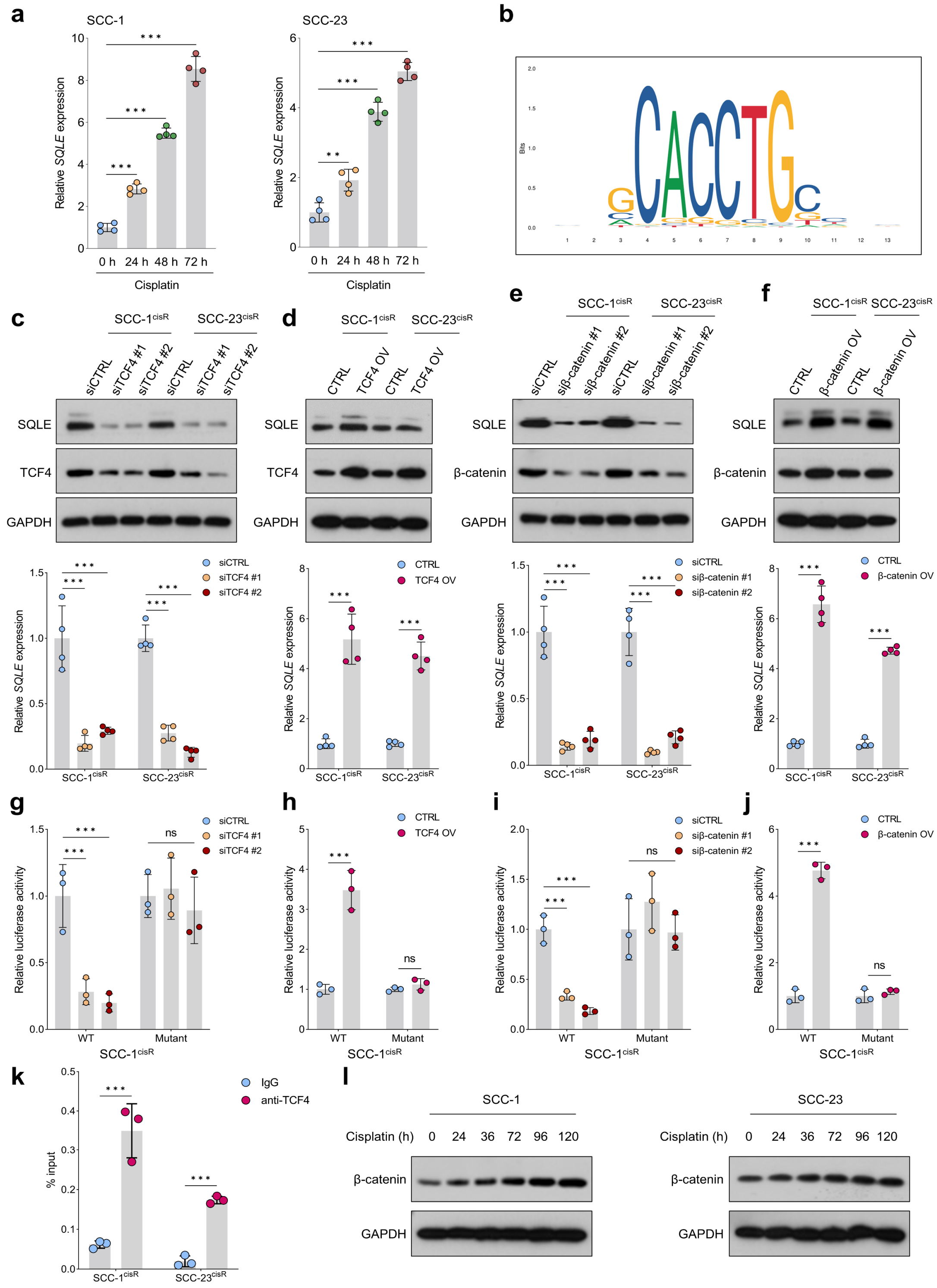

Figure S4. Transcriptional regulation of SQLE by the β-catenin/TCF4 complex in HNSCC cells. a) SQLE mRNA expression in SCC-1 and SCC-23 cells following cisplatin exposure for varying time durations (n=4). b) TCF4 binding motif sequence logo derived from the JASPAR database. c,d) SQLE expression in SCC-1^{cisR} and SCC-23^{cisR} cells after TCF4 depletion or overexpression (n=4). e,f) SQLE expression in SCC-1^{cisR} and SCC-23^{cisR} cells after β-catenin depletion or overexpression (n=4). g-j) Relative luciferase activities in indicated cells under specified treatments (n=3). k) ChIP-qPCR analysis of TCF4 enrichment in the SQLE promoter region (n=3). l) Dynamic expression of β-catenin in HNSCC cells following cisplatin exposure for indicated time durations. Data was presented as mean ± SD. ns (not significant), $^{**}p < 0.01$, $^{***}p < 0.001$ for Student's t-test and one-way ANOVA test.

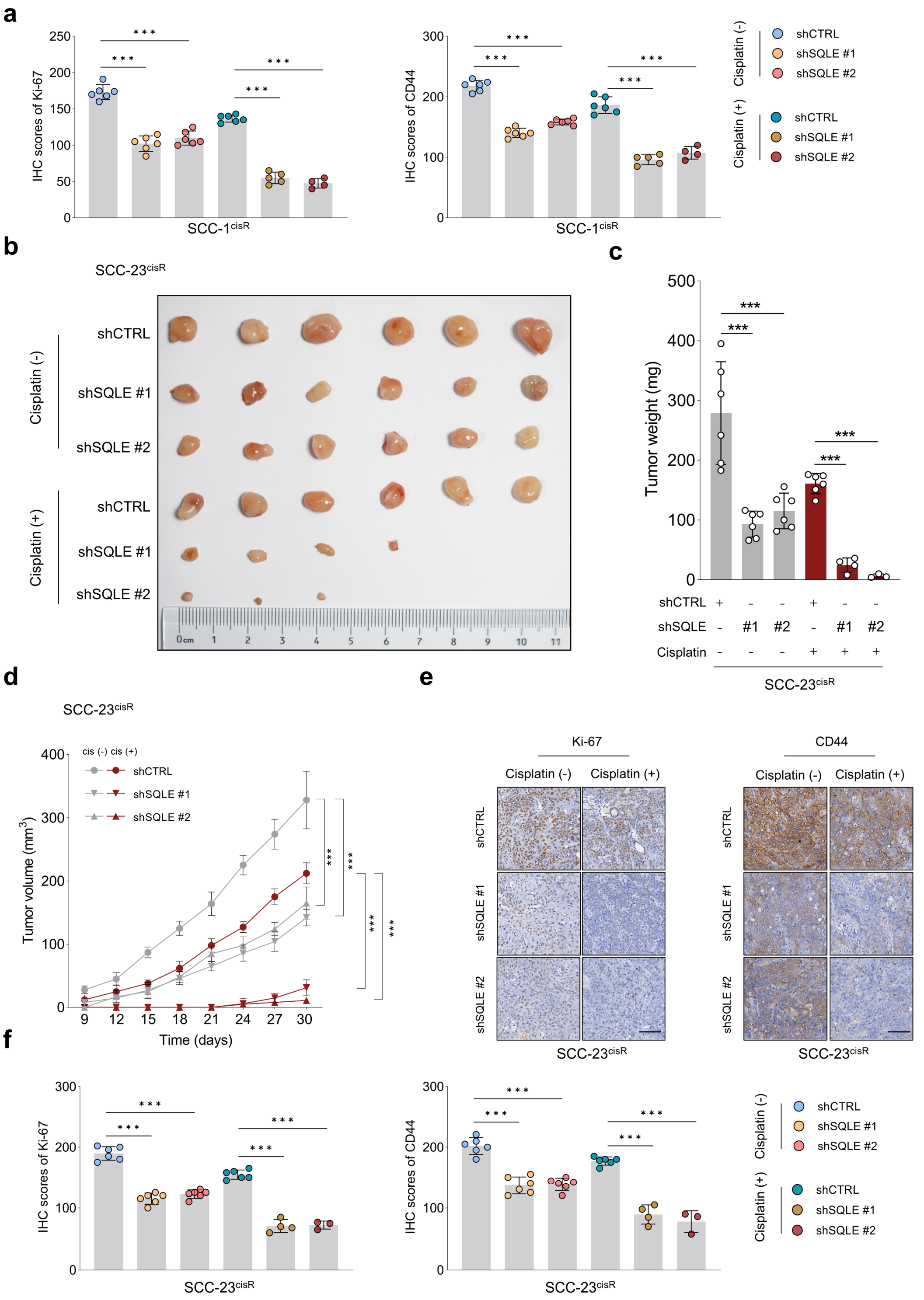

Figure S5. SQLE depletion enhanced cisplatin sensitivity in HNSCC cells in vivo. a) IHC scores of Ki-67 and CD44 in xenograft tumor tissues formed by SCC-1^{cisR} cells subjected to the indicated modifications (n=6). b-d) Effects of SQLE depletion on tumor size, weight, and volume in SCC-23^{cisR} cells with or without cisplatin treatment. Mice received either vehicle control or cisplatin (5 mg/kg intraperitoneally, twice per week) starting 1 week after tumor cell injection (n=6). e,f) IHC scores of Ki-67 and CD44 in xenograft tumor tissues formed by SCC-23^{cisR} cells under the specified treatments (n=6). Scale bar: 100 µm. Data was presented as mean ± SD. ***p < 0.001 for one-way ANOVA test.

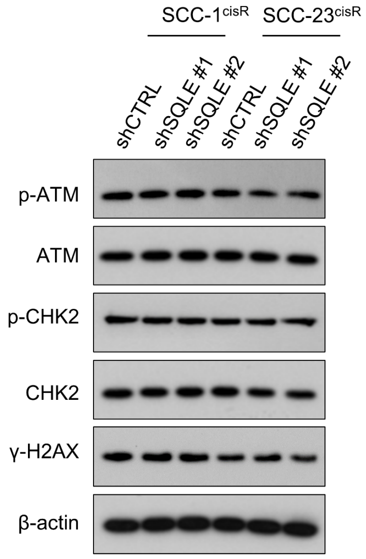

Figure S6. The alterations in the expression levels of p-ATM, ATM, p-CHK2, CHK2, and γ-H2AX following SQLE depletion in the SCC-1^{cisR} and SCC-23^{cisR} cell lines.

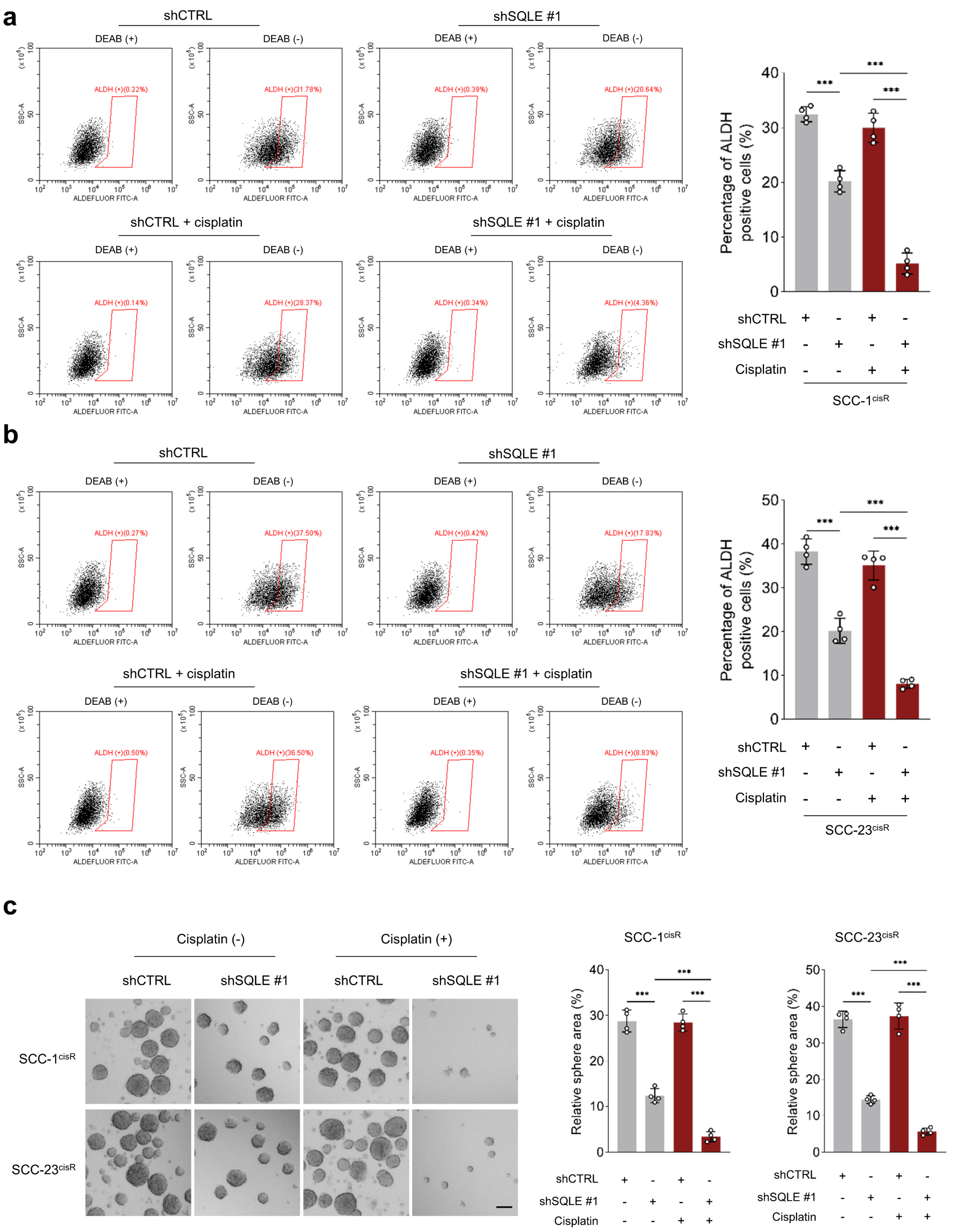

Figure S7. SQLE depletion diminished cancer stemness of HNSCC cells in vitro. a,b) Representative ALDEFLUOR™ assays and FACS analysis of control or SQLE-depleted SCC-1^{cisR} and SCC-23^{cisR} cells, with or without cisplatin treatment (n=4). c) Impact of SQLE knockdown on the sphere-forming ability of SCC-1^{cisR} and SCC-23^{cisR} cells in the presence or absence of cisplatin (n=4). Scale bar: 100 µm. Data was presented as mean ± SD. $***p < 0.001$ for one-way ANOVA test.

a

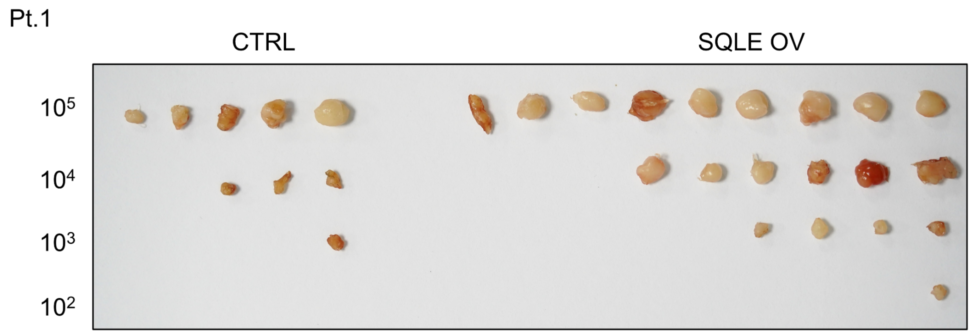

b

Cells injected	Tumor incidence/number of injection	
	CTRL	SQLE OV
10^5	5/10	9/10
10^4	3/10	6/10
10^3	1/10	4/10
10^2	0/10	1/10
CSC frequency	1/88807	1/15833
95% CI	1/183686-1/42936	1/35416-1/7079
P	9.37e-05	

Figure S8. SQLE overexpression promoted tumor initiation capacity of primary HNSCC cells in vivo. a) In vivo limiting dilution analysis of $EpCAM^{+}ALDH^{high}$ tumor cells from Pt. 1, infected with control and SQLE OV lentiviruses (n=10). The frequency of cancer cells capable of initiating tumors at each indicated cell dose was presented. b) Differences in CSC frequencies were calculated using the ELDA software.

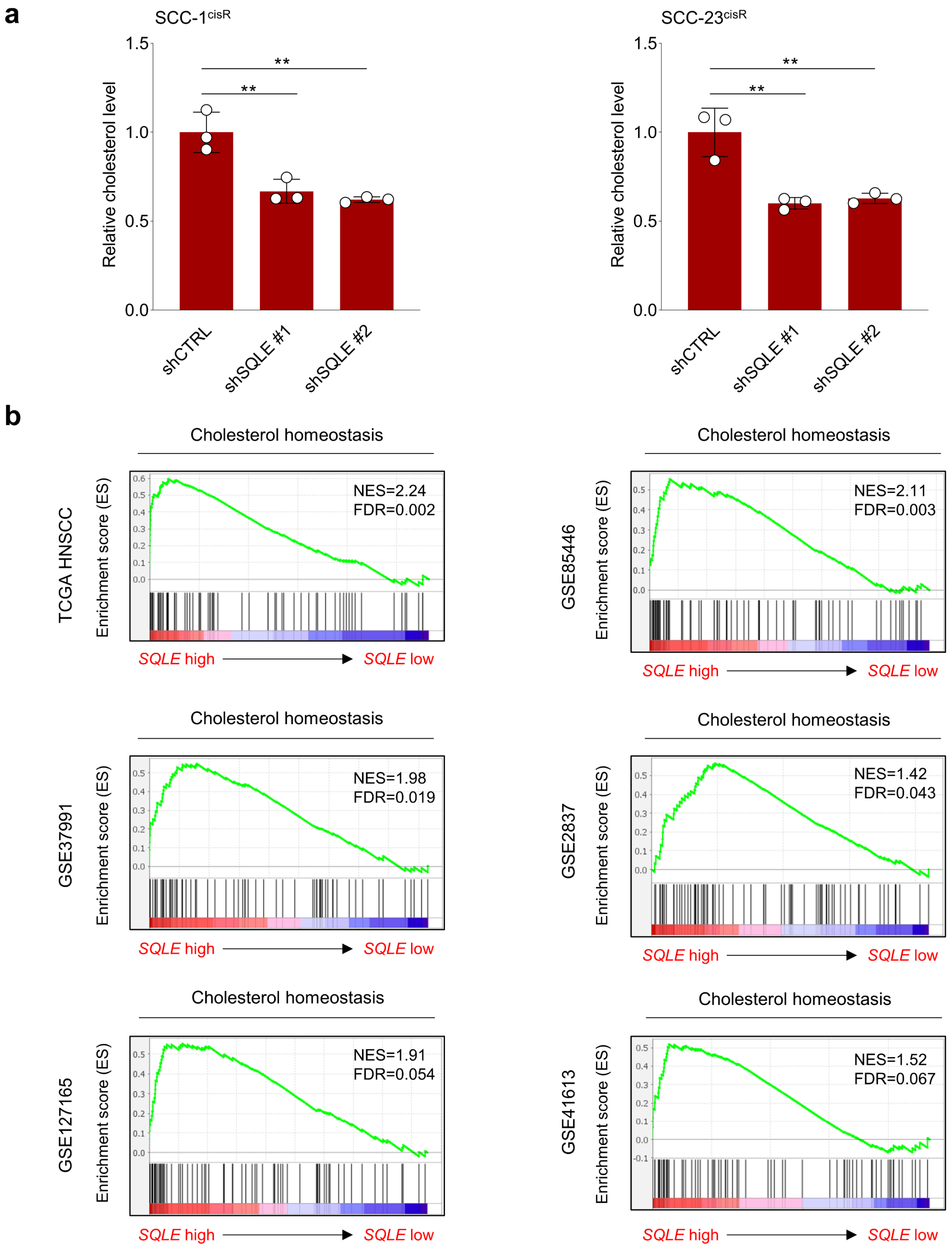

Figure S9. SQLE was essential for maintaining cholesterol homeostasis in HNSCC cells and tumor tissues. a) Cholesterol levels in SCC-1^{cisR} and SCC-23^{cisR} cells transfected with shSQLE #1, shSQLE #2, and shCTRL (n=3). b) GSEA demonstrates that genes involved in cholesterol homeostasis molecular signature were significantly enriched in *SQLE*-high HNSCC tumor tissues across multiple independent cohorts, including TCGA HNSCC, GSE85446, GSE37991, GSE2837, GSE127165, and GSE41613. Data was presented as mean ± SD. $^{**}p < 0.01$ for one-way ANOVA test.

a

Name	ES	NES	NOM p-val	FDR q-val
E2F_TARGETS	0.576	1.584	0.000	0.005
G2M_CHECKPOINT	0.571	1.567	0.000	0.003
PROTEIN_SECRETION	0.518	1.381	0.000	0.035
MYC_TARGETS_V1	0.498	1.374	0.000	0.033
MITOTIC_SPINDLE	0.499	1.368	0.000	0.030
UNFOLDED_PROTEIN_RESPONSE	0.470	1.264	0.015	0.088
HYPOXIA	0.454	1.242	0.007	0.107
IL2_STAT5_SIGNALING	0.434	1.191	0.020	0.212
INTERFERON_GAMMA_RESPONSE	0.429	1.172	0.034	0.248

b

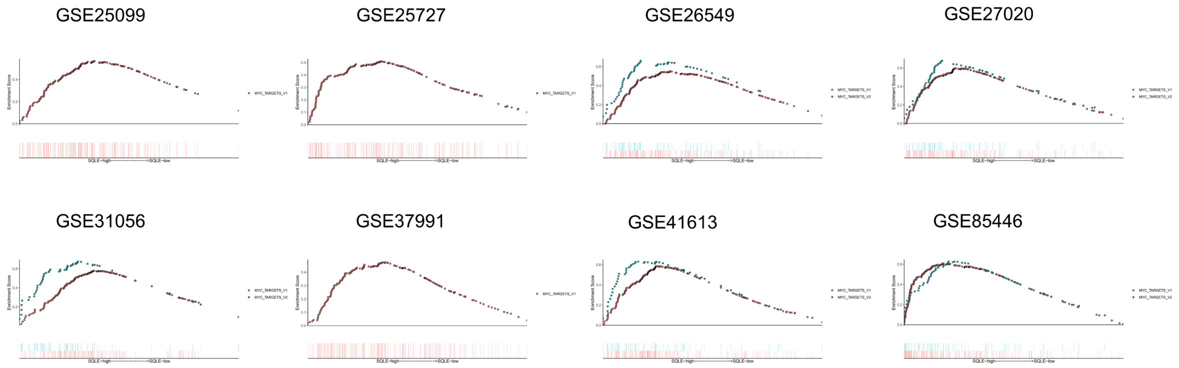

c

Dataset	Name	NES	FDR q-val
GSE25099	MYC_TARGETS_V1	1.716	0.242
GSE25727	MYC_TARGETS_V1	1.604	0.071
GSE26549	MYC_TARGETS_V2	1.900	0.018
	MYC_TARGETS_V1	1.692	0.061
GSE27020	MYC_TARGETS_V2	1.664	0.168
	MYC_TARGETS_V1	1.566	0.090
GSE31056	MYC_TARGETS_V1	1.644	0.069
	MYC_TARGETS_V2	1.474	0.089
GSE37991	MYC_TARGETS_V1	1.541	0.238
GSE41613	MYC_TARGETS_V1	1.854	0.002
	MYC_TARGETS_V2	1.610	0.068
GSE85446	MYC_TARGETS_V1	1.895	0.016
	MYC_TARGETS_V2	1.714	0.059

Figure S10. Strong association between SQLE expression and c-Myc target gene signatures in HNSCC. a) Top enriched signatures in the shCTRL group compared to the shSQLE group, as identified by GSEA analysis. b,c) The c-Myc target signatures MYC_TARGETS_V1 and/or MYC_TARGETS_V2 are enriched in the *SQLE*-high expression group across multiple HNSCC cohorts.

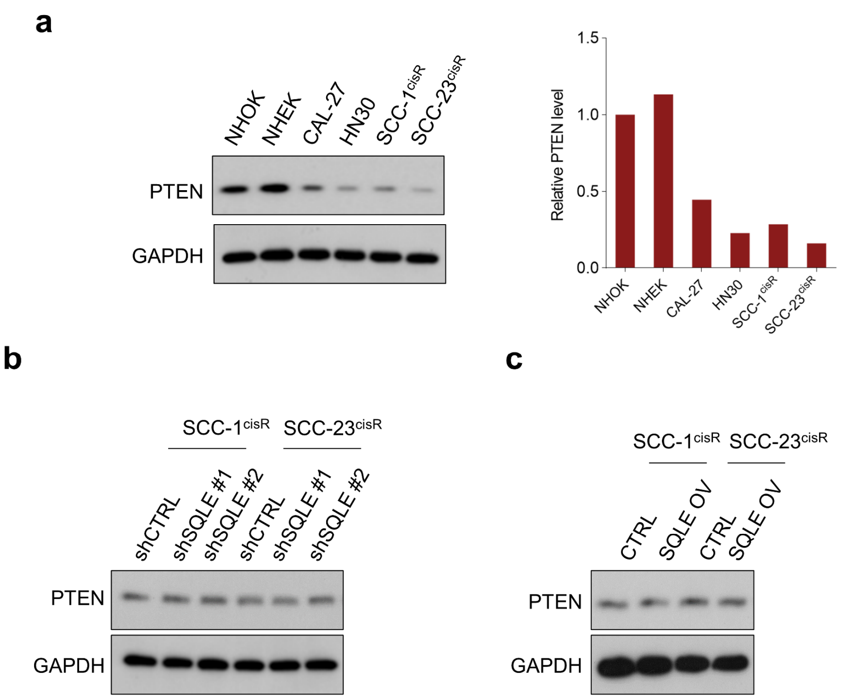

Figure S11. Influence of SQLE modifications on PTEN expression in SCC-1^{cisR} and SCC-23^{cisR} cells. a) PTEN expression levels in the specified cell lines. b,c) Effects of SQLE depletion or overexpression on PTEN expression in both SCC-1^{cisR} and SCC-23^{cisR} cells.

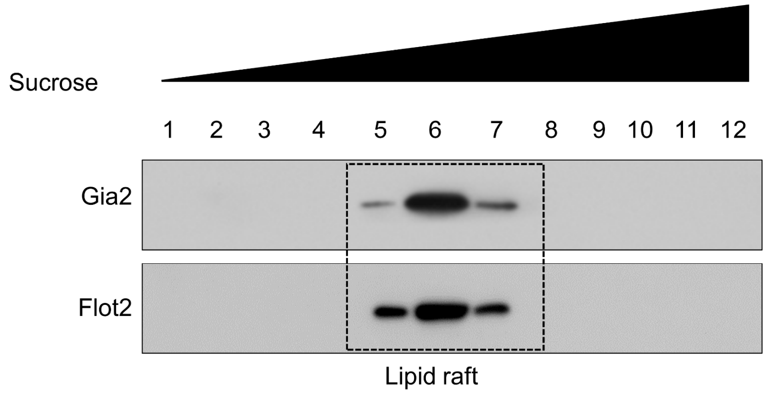

Figure S12. Western blotting analysis of the expression of Gia2 and Flot2 in the indicated fractions isolated with the sucrose gradient ultracentrifugation.

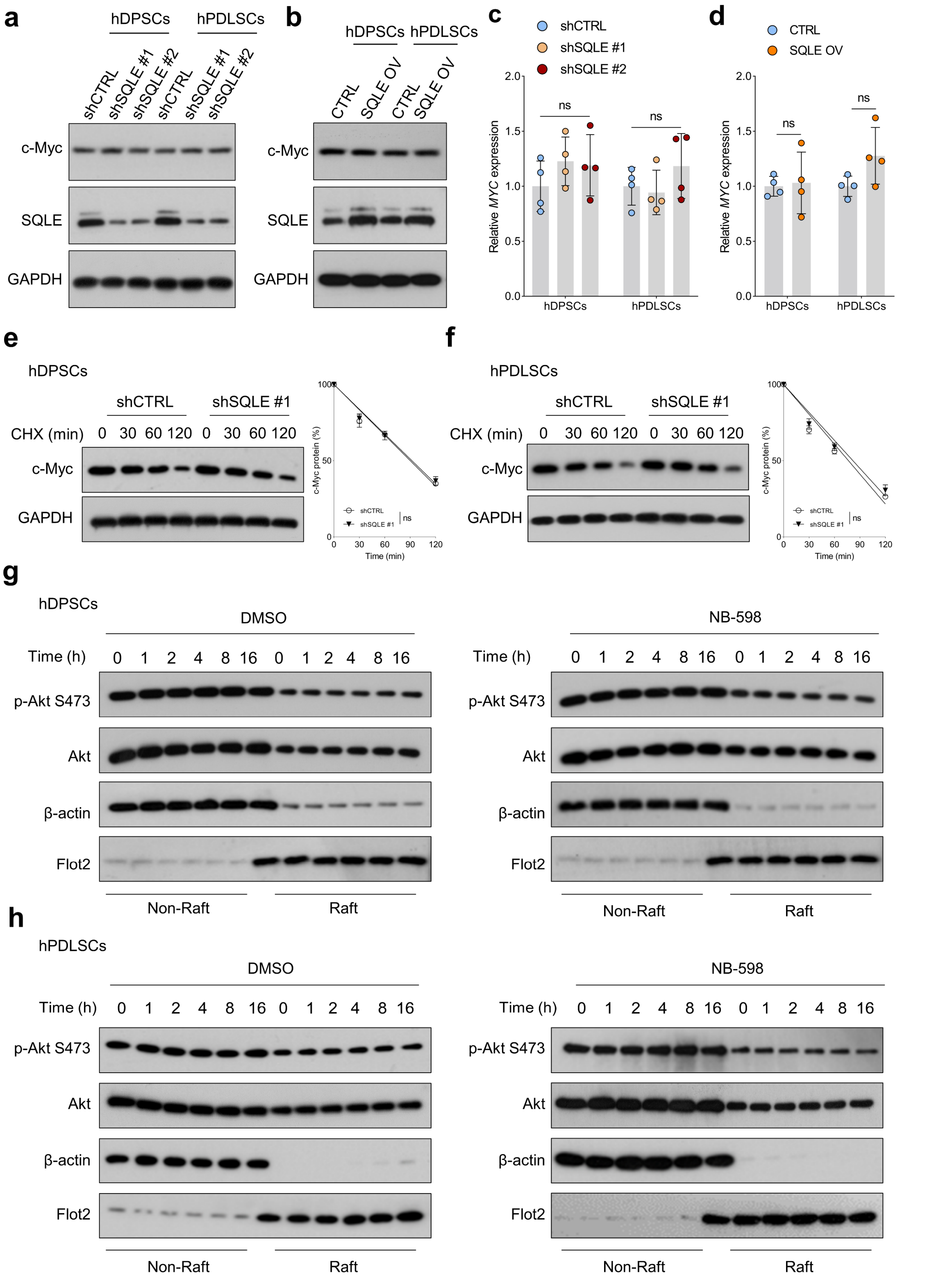

Figure S13. Effects of SQLE depletion on c-Myc stability and raft-localized Akt in human dental stem cells. a,b) Impact of SQLE depletion or overexpression on c-Myc protein expression in hDPSCs and hPDLSCs. c,d) Influence of SQLE depletion or overexpression on c-Myc mRNA expression in hDPSCs and hPDLSCs (n=4). e,f) CHX analysis illustrating the effect of SQLE depletion on c-Myc stability in hDPSCs and hPDLSCs. g,h) Impact of SQLE inhibition on the expression of raft-localized Akt in hDPSCs and hPDLSCs. Data was presented as mean ± SD. ns (not significant) for Student's t-test and one-way ANOVA test.

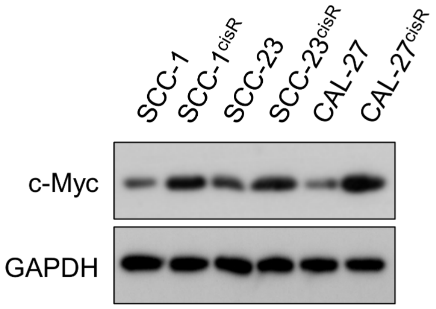

Figure S14. Western blot analysis of c-Myc expression in cisplatin-resistant cell lines and their parental cisplatin-sensitive counterparts.

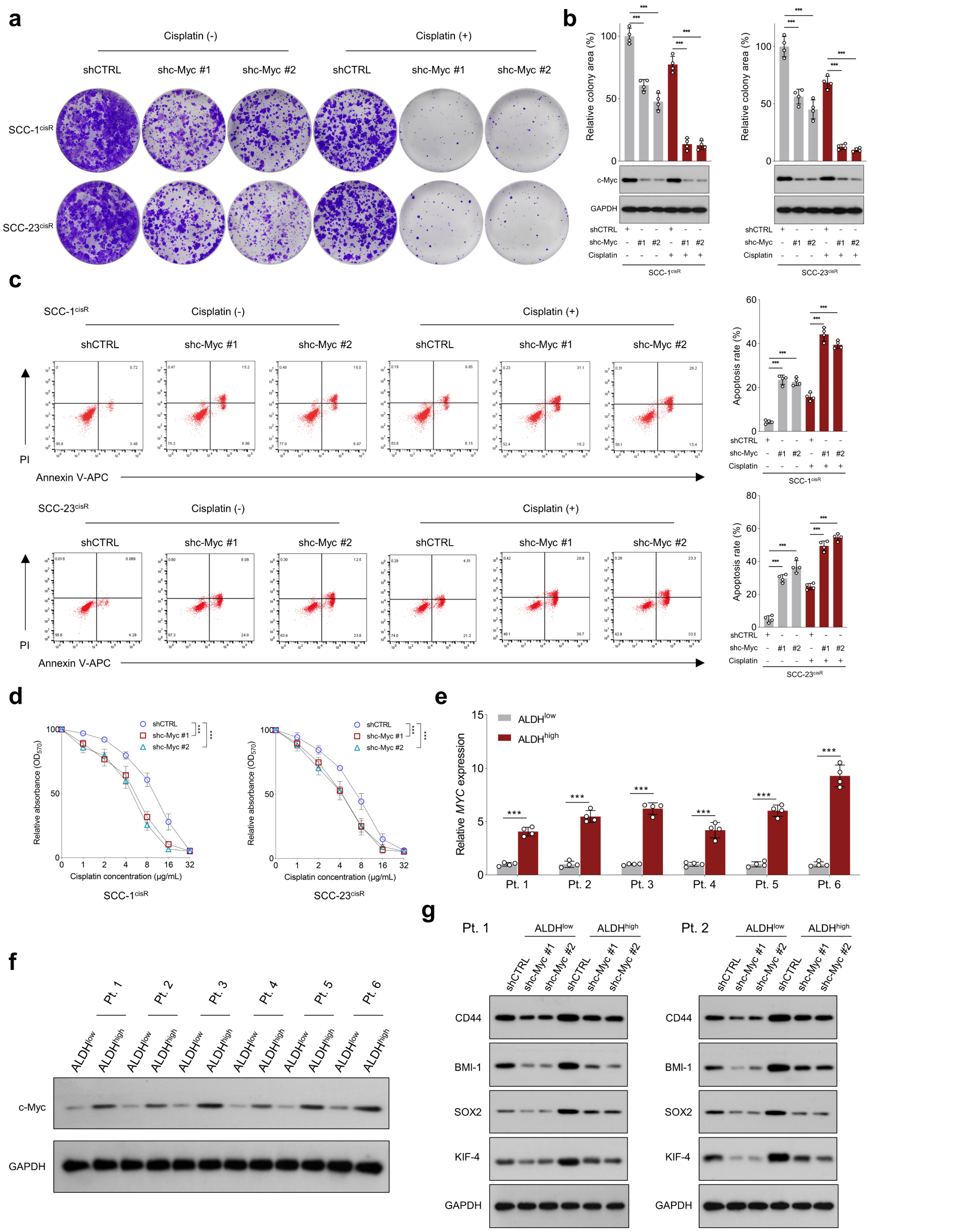

Figure S15. c-Myc depletion re-sensitizes cisplatin-resistant HNSCC cells to cisplatin and plays a critical role in regulating cancer stemness in HNSCC. a,b) Assessment of colony formation potential in SCC-1^{cisR} and SCC-23^{cisR} cells upon c-Myc depletion, with or without cisplatin treatment (n=4). c-Myc knockdown efficiency was evaluated by western blotting. c) Apoptosis rates in SCC-1^{cisR} and SCC-23^{cisR} cells following c-Myc depletion, in the presence or absence of cisplatin (n=4). d) Cell viability assessment in SCC-1^{cisR} and SCC-23^{cisR} cells subjected to c-Myc depletion and exposed to a gradient of cisplatin concentrations (n=4). e,f) Analysis of c-Myc mRNA and protein expression in ALDH^{high} and ALDH^{low} cells isolated from six HNSCC patients (n=4). g) Western blotting examination of the impact of c-Myc depletion on CD44, BMI-1, SOX2, and KIF-4 expression in ALDH^{high} and ALDH^{low} primary HNSCC cells, obtained from HNSCC patient case #1 (Pt. 1) and case #2 (Pt. 2). Data was presented as mean ± SD. ***$p < 0.001$ for one-way ANOVA test.

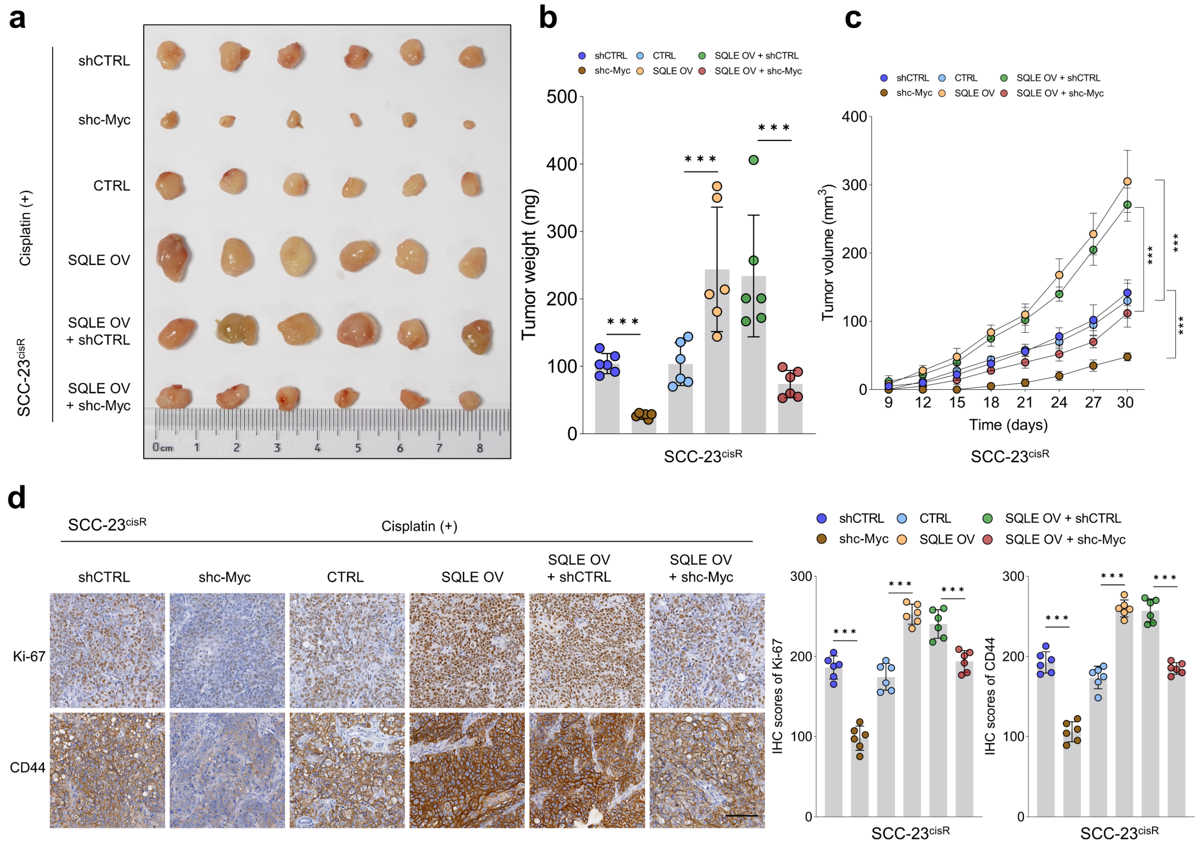

Figure S16. c-Myc serves as a functional downstream target of SQLE. a-c) Effects of c-Myc knockdown on tumor growth in SQLE-overexpressing SCC-23^{cisR} cells (n=6). d) IHC scores of Ki-67 and CD44 in xenograft tumor tissues subjected to the indicated treatments (n=6). Scale bar: 100 µm. Data was presented as mean ± SD. ***$p < 0.001$ for one-way ANOVA test.

a

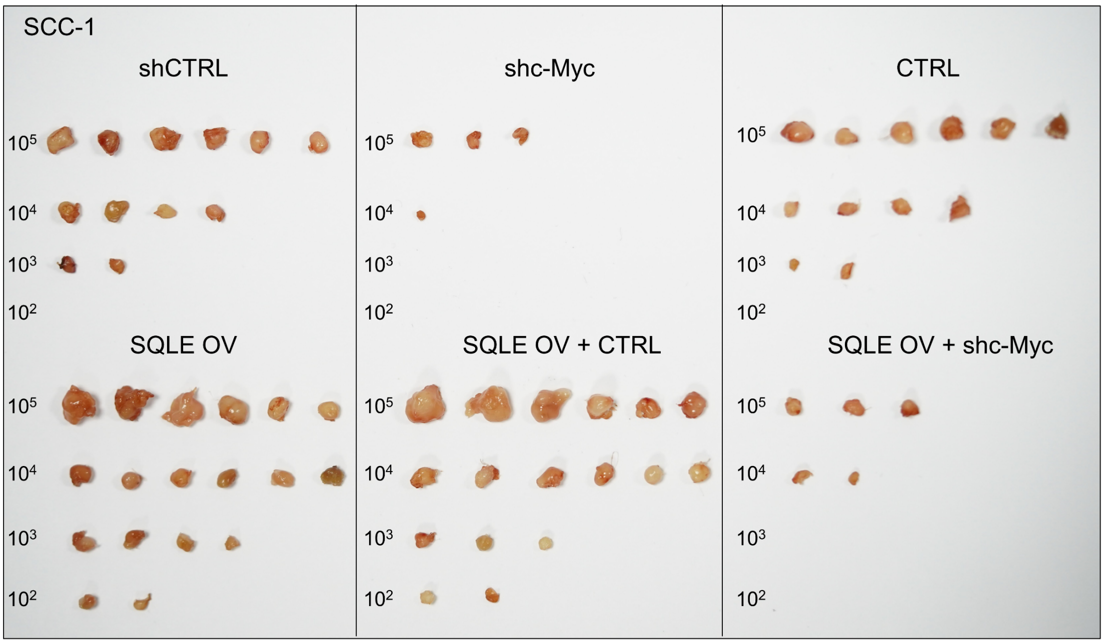

b

Cells injected	Tumor incidence/number of injection					
	shCTRL	shc-Myc	CTRL	SQLE OV	SQLE OV + CTRL	SQLE OV + shc-Myc
10^5	6/6	3/6	6/6	6/6	6/6	3/6
10^4	4/6	1/6	4/6	6/6	6/6	2/6
10^3	2/6	0/6	2/6	4/6	3/6	0/6
10^2	0/6	0/6	0/6	2/6	2/6	0/6
CSC frequency	1/6808	1/122851	1/6808	1/670	1/948	1/96178
95% CI	1/16690-1/2777	1/341022-1/44257	1/16690-1/2777	1/1653-1/272	1/2470-1/364	1/250059-1/36993

c

Group differences in CSC frequencies	*P* value
shCTRL *vs.* shc-Myc	1.53e-05
CTRL *vs.* SQLE OV	2.96e-04
SQLE OV + CTRL *vs.* SQLE OV + shc-Myc	2.12e-12

Figure S17. c-Myc functions as a downstream target of SQLE, critical for maintaining cancer stemness in HNSCC. a) In vivo limiting dilution analysis of SCC-1 cells subjected to indicated treatments (n=6), demonstrating the frequency of tumor cells capable of initiating xenografts at each injected cell dose. b,c) Differences in CSC frequencies between the indicated groups were calculated using ELDA software.

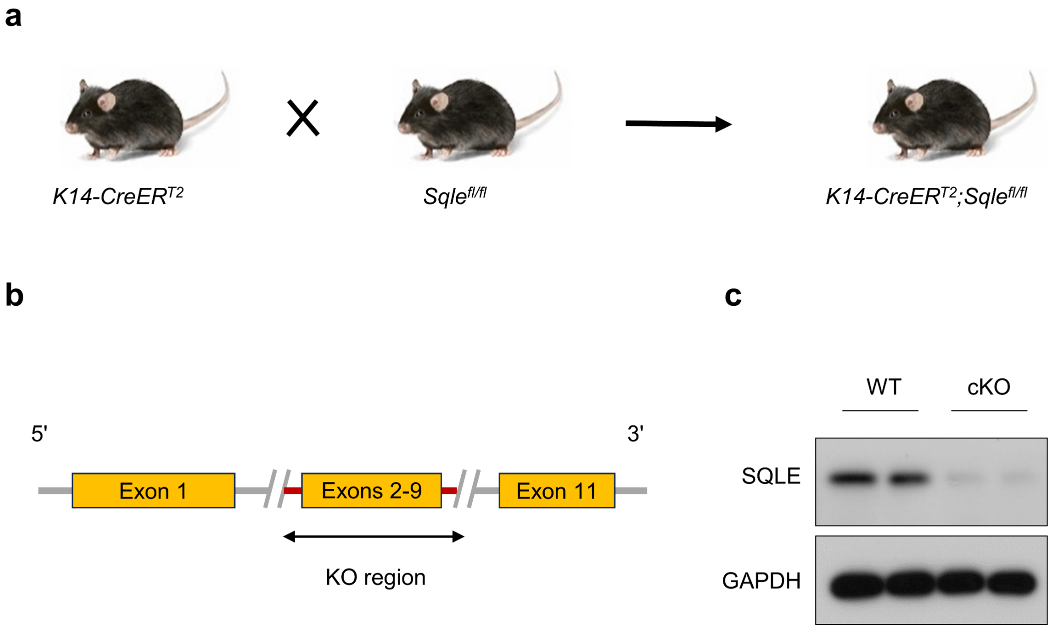

Figure S18. Generation of *Sqle* cKO mouse model. a,b) Schematic diagram illustrating the generation of the *Sqle* cKO mouse model. c) Western blot analysis of SQLE expression in tongue tissue from *Sqle*^{CTRL} and *Sqle*^{cKO} mice.

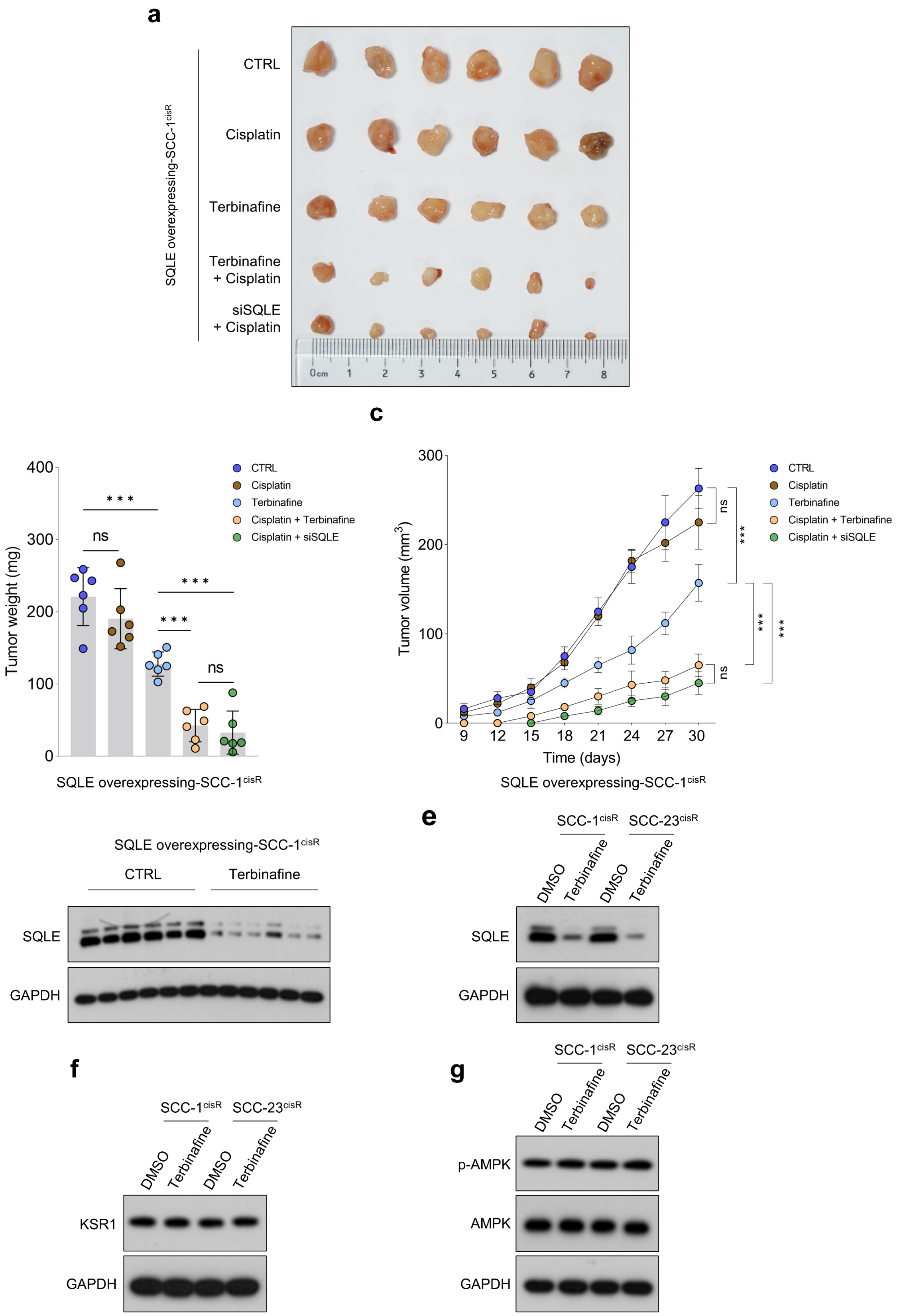

Figure S19. Terbinafine suppressed the tumorigenesis of HNSCC cells by targeting SQLE. a-c) Tumor size, weight, and volume in the indicated treatment groups (n=6). d) SQLE expression in terbinafine-treated xenografts. e) SQLE expression in HNSCC cells treated with terbinafine compared to DMSO-treated control cells. f-g) Expression levels of KSR1, p-AMPK, and AMPK in HNSCC cells treated with terbinafine compared to DMSO-treated control cells. Data was presented as mean ± SD. ns (not significant), ***$p < 0.001$ for one-way ANOVA test.

Table S1. The clinicopathological information of in-house HNSCC cohort.

Clinicopathological parameters	Number of patients (n, %)
**Age**	
>=60	45 (44.12%)
<60	57 (55.88%)
**Gender**	
Male	67 (65.69%)
Female	35 (34.31%)
**Smoking**	
Yes	53 (51.96%)
No	49 (48.04%)
**Alcohol intake**	
Yes	36 (35.29%)
No	66 (64.71%)
**HPV infection status**	
Negative	81 (79.41%)
Positive	21 (20.59%)
**TNM stage**	
I-II	57 (55.88%)
III-IV	45 (44.12%)
**Differentiation**	
G1	65 (63.73%)
G2-G3	37 (36.27%)

Table S2. Sequences of primers and oligos used in this study.

Gene	Sequence (5′-3′)
SQLE	F: CTTCTCCTCAAAGCGAGCACA
	R: TTATTTAAAAATCGCCTGCTGGA
MYC	F: CTGGTGCTCCATGAGGAGA
	R: CCTGCCTCTTTTCCACAGAA
LDHA	F: ATCTTGACCTACGTGGCTTGGA
	R: CCATACAGGCACACTGGAATCTC
CDK2	F: TCTGCCATTCTCATCGGGTC
	R: ATTTGCAGCCCAGGAGGATT
CCNA2	F: TGGAAAGCAAACAGTAAACAGCC
	R: GGGCATCTTCACGCTCTATTT
PCNA	F: CCTGCTGGGATATTAGCTCCA
	R: CAGCGGTAGGTGTCGAAGC
EIF4E	F: AACAAACGGGGAGGACGATG
	R: CCAAAAGCGATCGAGGTCAC
ENO1	F: AAAGCTGGTGCCGTTGAGAA
	R: GGTTGTGGTAAACCTCTGCTC
HSPD1	F: CGGCCGGCTTAGTCTAGTT
	R: ATTTGACCCTTGAGCCGTAG
CCND2	F: TTCCCTCTGGCCATGAATTAC
	R: GGGCTGGTCTCTTTGAGTTT
PCK2	F: CTCCACAACCTCCAACCATCTT
	R: TTGCCTGGGACTGGAAACTG
GAPDH	F: TGCACCACCAACTGCTTAGC
	R: GGCATGGACTGTGGTCATGAG
siSQLE	GCACCACAGTTTAAAGCAA
shSQLE #1	GCAGCCGGGTGGTTATCATGT
shSQLE #2	GGGAGTTCAGTACAAGGATAA
